# Analysis of benzene air quality standards, monitoring methods and concentrations in indoor and outdoor environment

**DOI:** 10.1016/j.heliyon.2019.e02918

**Published:** 2019-11-29

**Authors:** Abinaya Sekar, George K. Varghese, M.K. Ravi Varma

**Affiliations:** a Department of Civil Engineering, Environmental Engineering Lab, National Institute of Technology Calicut, 673601, India; b Department of Physics, Applied Optics and Instrumentation Lab, National Institute of Technology Calicut, 673601, India

**Keywords:** Civil engineering, Environmental analysis, Environmental chemistry, Environmental engineering, Environmental hazard, Environmental health, Environmental management, Environmental pollution, Environmental risk assessment, Environmental toxicology, Air pollution, Benzene, Exposure, Global air quality standards, Risk assessment

## Abstract

Benzene is a proven carcinogen. Its synergistic action with other pollutants can damage different components of the biosphere. Literature comparing the air quality standards of benzene, its monitoring methods and global concentrations are sparse. This study compiles the worldwide available air quality standards for benzene and highlights the importance of strict and uniform standards all over the world. It was found that out of the 193 United Nation member states, only 53 countries, including the European Union member states, have ambient air quality standard for benzene. Even where standards were available, in most cases, they were not protective of public health. An extensive literature review was conducted to compile the available monitoring and analysis methods for benzene, and found that the most preferred method, i.e, analyzing by Gas Chromatography and Mass spectroscopy is not cost effective and not suitable for real-time continuous monitoring. The study compared the concentrations of benzene in the indoor and outdoor air reported from different countries. Though the higher concentrations of benzene noticed in the survey were mostly from Asian countries, both in the case of indoor and outdoor air, the concentrations were not statistically different across the various continents. Based on the analyzed data, the average benzene level in the ambient air of Asian countries (371 μg/m^3^) was approximately 3.5 times higher than the indoor benzene levels (111 μg/m^3^). Similarly, the outdoor to the indoor ratio of benzene level in European and North American Countries were found to be 1.2 and 7.7, respectively. This compilation will help the policymakers to include/revise the standards for benzene in future air quality guideline amendments.

## 1 Introduction

### 1.1 General

Volatile organic compounds (VOCs) are generally defined by the physicochemical properties like vapor pressure, molecular structure, air/water partition coefficient and boiling point. American Society for Testing and Material have defined VOCs by vapor pressure; “VOCs are organic compounds that have vapor pressure greater than 0.0133 kPa at 298 K” (American Society for Testing and Materials, 1996). The European Union have also defined VOCs with respect to vapor pressure; “VOCs must have a minimum vapor pressure of 0.01 kPa at 293 K” (European Union, 1999). World Health Organization (WHO) have defined VOCs with respect to boiling point; Very volatile organic compounds (VVOCs) have boiling points in the range of <0 to 50–100 C, Semi-volatile organic compounds (SVOCs) have it in the range of 240–260 °C to 380–400 °C and the Volatile organic compounds (VOCs) have boiling points in the range of 50–100 °C to 240–260 °C (World Health Organization, 1989). The VOCs emitted in the atmosphere include saturated and unsaturated hydrocarbons, organic alcohols, aromatic hydrocarbons, halogenated organic compounds and sulfur compounds (Keller, 1988). Out of these, organic compounds like benzene, toluene, ethylbenzene and xylene, commonly called as BTEX compounds, are found to be higher in the ambient air (Gaur et al., 2016; Montero-Montoya et al., 2018; Tiwari et al., 2010). Among the BTEX compounds, Benzene demands special attention. The US EPA risk assessment guidelines of 1986 had classified benzene as a “known human carcinogen” (Category A) (USEPA, 1986). The current carcinogenic risk assessment guidelines given by US EPA in 2005 has characterized benzene as a known human carcinogen based on human exposure evidence along with other supporting evidence from animal studies. Occupational based human exposure studies have concluded that exposure to benzene leads to toxic effects, both by oral and inhalation exposure (USEPA, 2005). Considering the toxic profile and the ubiquitous nature, it is necessary to monitor and regulate benzene in the ambient air.

### 1.2 Properties of benzene

Benzene remains in the vapor phase in the air. The lifetime of benzene in air ranges from a few hours to days and is dependent on the environmental conditions and the presence of other pollutants. The most important mode of degradation of benzene in the environment is through oxidation by hydroxyl radicle and subsequent removal by rain (WHO Regional Office for Europe, 2000). The physicochemical properties of benzene are shown in Table 1.

**Table 1:** Physico-chemical properties of benzene.

Parameter	Value/Nature
Chemical formula	C_6_H_6_
Molecular weight	78.11 g/mol
Nature	Volatile, Colorless, highly flammable
Odor	Sweet with an odor threshold of 1.5 ppm
Vapor pressure	95.2 mm Hg at 25 °C
Octanol/water partition coefficient (K_ow_)	2.13
Conversion factors in gaseous form	1 ppm = 3.19 mg/m^3^ 1 mg/m^3^ = 0.313 ppm

### 1.3 Sources of benzene in ambient air

Sources of benzene can be both natural and anthropogenic. Natural sources include emission from volcanoes and forest fires. Anthropogenic sources include emission from crude oil, gasoline and industrial processes. Benzene is used in the manufacturing of plastics, resins, synthetic fibers, rubbers, dyes, detergents, drugs, pesticides, etc. and as a lubricant. One of the most widespread sources of benzene in indoor air is cigarette smoke and it was found that the median level of benzene in the homes of smokers was higher than in the homes of non-smokers. On examining human exposure to benzene, it was found that smokers exhale around 14 μg/m^3^ and non-smokers, around 2 μg/m^3^ of benzene (Wallace, 1989). Studies conducted in the United States show that, at the national level, half of the total exposures to benzene happened through tobacco smoke. In countries like India where biomass is burned using traditional methods to meet the domestic energy requirements, the indoor concentrations of benzene were found to be significantly high (Sinha et al., 2006, 2005). Other sources of benzene included vehicle exhaust, evaporation from motor vehicles and petroleum retails outlets while storing and distributing petrol (ATSDR, 2007a).

Benzene is also emitted into the atmosphere during its production. In 1988, the worldwide production of benzene was 20 million tones and the production was increasing every year after that (Merchant Research and Consulting, 2014). In 2012 the production increased to 42.9 million tones (WHO Regional Office for Europe, 2000) and the benzene market in 2021 is projected to be worth more than US$69 billion (Ceresana market research, 2015). Benzene demand is high for the production of ethylbenzene, Cumene, Cyclohexane and aniline. China, The United States and Western Europe are the highest consumers of benzene (Merchant Research and Consulting, 2014).

### 1.4 Benzene pathway and health effects

The entry of benzene into the human body takes place via lungs, gastrointestinal tract and through the skin. About half of the benzene inhaled passes through the lungs and enter the bloodstream. Once it enters the bloodstream it undergoes primary oxidative metabolism in the liver by cytochrome P-450 2E1 system. The major metabolites of the above process are phenol, catechol and hydroquinone which are stored temporarily in the bone marrow. Phenol is the major metabolite of the process and it is eliminated in urine as sulfate and glucuronide conjugate. Exposure to benzene has a clear association with acute nonlymphocytic leukemia, chronic nonlymphocytic leukemia and chronic lymphocytic leukemia (ATSDR, 2007b; Bayliss et al., 1998; US EPA Integrated Risk Information system, 1999). Other than cancer risk, there are also some identified non-cancer risks like headache, dizziness, drowsiness, confusion, tremors and loss of consciousness, moderate eye irritation and skin irritation. When the exposure is combined with alcohol consumption, the effect of toxicity is higher (World Health Organization, 1993).

The objective of the paper is to collect the available ambient air quality standards for benzene worldwide and to analyze the monitoring methods for benzene in air. Due to its known carcinogenic effect, it is highly important to know its concentration in the ambient air through accurate real-time monitoring. A compilation of the ambient air quality standards for benzene in different countries and a critical analysis of its adequacy to protect human health will help the policymakers to include/revise the standards for benzene in future air quality guideline amendments. Irrespective of the economic status of the country stringent standard should be put for benzene in order to protect human health. Also, there is a need to eliminate heterogeneity of air quality standards around the globe as air pollution does not respect political boundaries. This is perhaps the first study to report the worldwide ambient air quality standard for benzene, its adequacy in protecting human health and the common methods adopted for monitoring.

**Figure 1:**
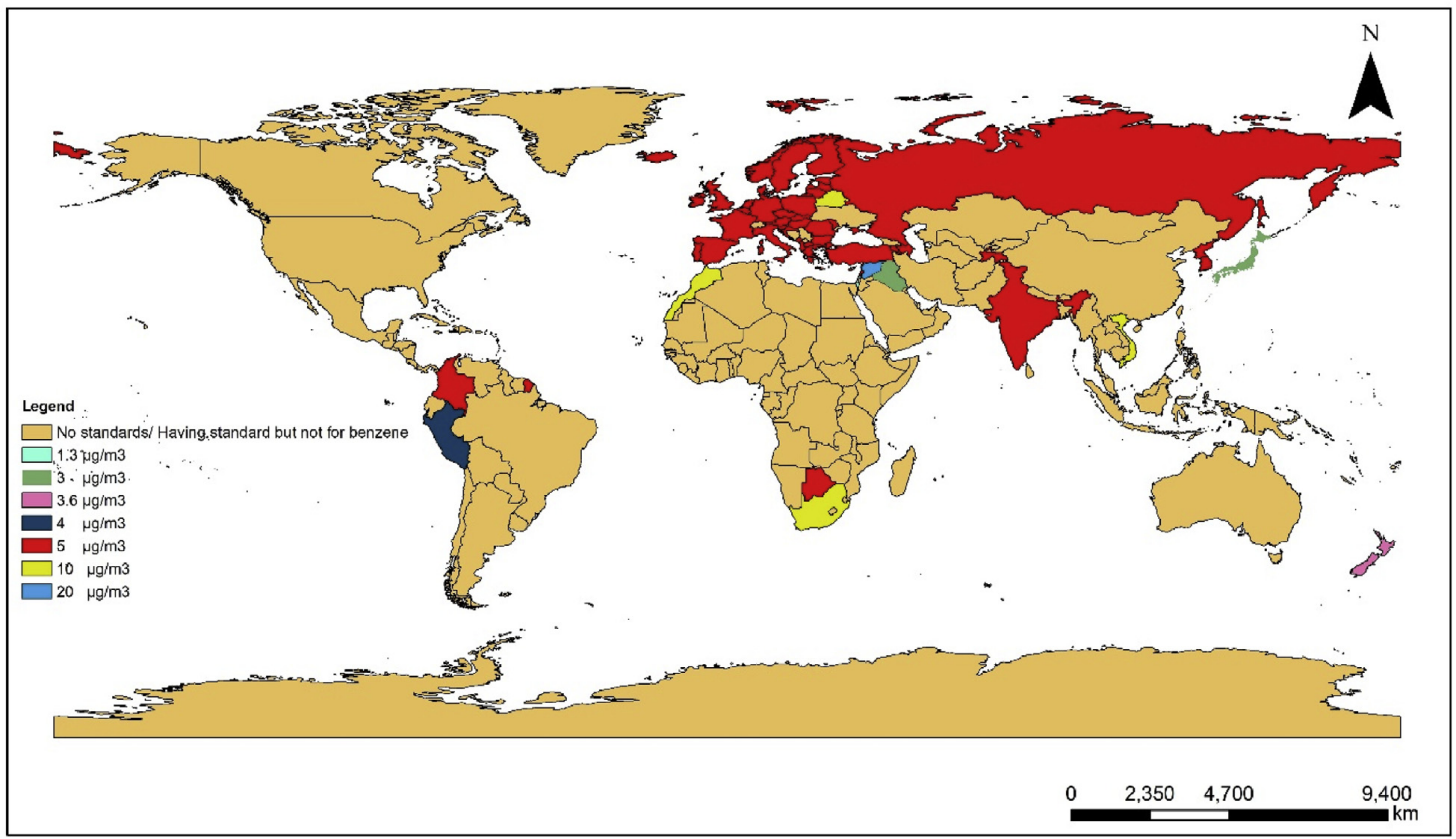
Map representing worldwide standards for benzene.

Efforts were put to obtain the latest standards of benzene in ambient air for all the countries directly from the government repositories. The details of these standards are given as supplementary material. But, in a few cases, peer-reviewed journals and online resources were the only source available for the information and may not reflect the latest standards (see Figure 1).

## 2 Standards for benzene in the ambient air quality standards of various countries

### 2.1 Asia

From the scientific literatures reviewed and the details collected from the government repositories, it was found that out of the 48 Asian countries, only 14 have standards for benzene. Armenia, Azerbaijan and Tajikistan follow Russian air quality standards and North Korea, Turkey follow EU standards. Remaining 10 countries, which include India, Iraq, Japan, Lebanon, Russia, South Korea, Syria, Isreal and Vietnam have their own standards for benzene. In the case of Vietnam, the standard for benzene is prescribed in National Technical Regulation on Hazardous substance in Ambient Air (QCVN 06: 2009/BTNMT), with the maximum allowable limit of 22 μg/cm^3^ on hourly basis and 10 μg/cm^3^ annually (Clean Air Initiative for Asian Cities (CAI-Asia) Center, 2010). Lebanon follows similar to European Union limit of 5 μg/m^3^ (European Union, 2016). In South Korean ambient air quality standards, the permissible limit for benzene is 5 μg/m^3^ (Annual) (Ministry for the Environment South Korea, 2010). The prescribed limit for benzene in the air is 5 μg/m^3^ (Annual) in the case of India (Central Pollution Control Board, 2009). As per the Russian standards the maximum allowable concentration (MAC) is 100 μg/m^3^ (24 h) and according to the standard for sanitary protection zone (GN 2.1.6.1338–03) the permissible level for benzene is 0.3, 0.1, 0.005 mg/m^3^ for the averaging time of 20 min, 24 h and one year respectively (ENVIRON, 2014). Japan's air quality standard specifies a limit of 3 μg/m^3^ (annual) which is stringent compared to EU standards (Ministry of Environment Japan, 2009). In Iraq and Syria, the ambient air quality standard for benzene is 0.003 mg/m^3^ (annual) and 20 μg/m^3^ (annual), respectively (Ministry of Oil, 2018; Offical Gazetta, 2003). Israeli standards are the most stringent among all, with limits set at 3.9 μg/m^3^ (24 h) and 1.3 μg/m^3^ (annual) (Department of Environmental health, 2017).

### 2.2 Australia

Among 14 countries in the Australian continent, from the available sources of information, only 6 countries have got its own ambient air quality standards. In that, only New Zealand prescribes a standard for benzene, which is 5 μg/m^3^ (Annual). The establishment of their standard is based on EU and UK approaches (Ministry for the Environment and the Ministry of Health, 2002).

### 2.3 Africa

In the African continent, the standard for benzene is present in the Botswana Bureau of Standards, Morocco air quality standard and the South African ambient air quality standards. The permissible limit for benzene in Botswana standard is 5 μg/m^3^ (annual) (Modupe O. Akinola, 2017). 10 μg/m^3^ (calendar year) is the limit set for human health protection in the Moroccan (Chirmata et al., 2017) & South African standard (Department of Environmental Affairs South Africa, 2009). Air quality standards of Kenya specify the limits for Non-methane volatile organic compounds (instant peak 700 ppb) and Total volatile organic compounds (600 μg/m^3^-24 h), but not explicitly for benzene (Ministry for Environment and Mineral Resources, 1999).

### 2.4 Europe

It is suggested that all the 28-member states in Europe should comply with the limit set for benzene at 5 μg/m^3^ (annual) as per the Directive 2008/50/EC on ambient air quality and cleaner air for Europe. Among the European Union countries, France has the lowest long-term objective limit for benzene at 2 μg/m^3^ (Annual) (Air quality observatory in the Paris region, 2018). Scotland and Northern Ireland set out an objective value of 3.25 μg/m^3^ (Air Pollution Information System, 2016), Sweden and Malta have a standard for annual mean with upper threshold: 3.5 μg/m^3^ and lower threshold of 2 μg/m^3^ (Ambient Air Quality Standards, 2010; Swedish Code of Statutes, 2010). Among countries of Europe other than the EU member states, Albania has a permissible limit of 5 μg/m^3^-8h in primary and secondary standards (Environmental center for Administration and Technology, 2008) and Belarus has limits 10 μg/m^3^ (calendar year) and 40 μg/m^3^ (24h) (European Union, 2012). Certain countries like Moldova and Ukraine follows standards of the Russian Federation with a maximum allowable concentration of 100 μg/m^3^ (24 h) (European Union, 2012).

### 2.5 North & South America

Among the 33 countries in the North and South America, only Colombia and Peru have got limits for benzene, with the allowable value of 5 μg/m^3^ (annual) (Airlex worldwide Air quality legislation, 2013) and 4 μg/m^3^ (calendar year) (Decreto Supremo N 074, 2001), respectively, in their air quality standards. In case of Cuba the Maximum allowable concentration for 20 min is 1 mg/m^3^ (Airlex worldwide Air quality legislation, 2013). Tables 2 and 3 summarizes the limit set for benzene in the air quality standards of different countries.

**Table 2:** Worldwide ambient air quality standards for benzene.

Continent	Country	Limit (μg/m^3^)	Averaging interval	Standard/Definitions	Reference
Asia	India	5	Annual	Air quality standards	(Central Pollution Control Board, 2009)
Asia	Iraq	3	Annual	Air quality standards	(Ministry of Oil, 2018)
Asia	Japan	3	Annual	Air quality standards	(Ministry of Environment, 2009)
Asia	Lebanon	5	Annual	Limit value	(European Union, 2016)
Asia	Russia	100 300 5	24 h 20 min Annual	Maximum Allowable Concentration GN 2.1.6.1338–03 for sanitary protection zone	(ENVIRON, 2014)
Asia	South Korea	5	Annual	Air quality standard	(Ministry for the Environment South Korea, 2010)
Asia	Syria	20	Annual'	Air quality standard	(Offical Gazetta, 2003)
Asia	Vietnam	22 10	1 h Annual	National Technical Regulation on Hazardous Substances in Ambient Air (QCVN 06:2009/BTNMT)	(Clean Air Initiative for Asian Cities (CAI-Asia) Center, 2010)
Asia	Israel	3.9 1.3	24 h Annual	Air quality standards	(Department of Environmental health, 2017)
Australia	New Zealand	5 (2002) 3.6 (2010)	Annual	Air quality standards	(Ministry for the Environment and the Ministry of Health, 2002)
Africa	Botswana	5	Annual	Air quality standards	(Modupe O. Akinola, 2017)
Africa	Morocco	10	Annual	Air quality standards	(Chirmata et al., 2017)
Africa	South Africa	10	Annual	Air quality standards	(Department of Environmental Affairs South Africa, 2009)
Europe	European Union	5	Annual	The limit value for human health protection	(European Union, 2008)
Europe	France	2	Annual	Long-term objective	(Air quality observatory in the Paris region, 2018)
Europe	Albania	5	8 h	Primary and secondary standards	(Environmental center for Administration and Technology, 2008)
Europe	Belarus	40 10	24 h Annual	Maximum allowable concentration	(European Union, 2012)
Europe	Sweden	Upper threshold: 3.5 Lower threshold: 2	Annual	Environmental quality standards	(Swedish Code of Statutes, 2010)
Europe	Malta	Upper threshold: 3.5 Lower threshold: 2	Annual	Ambient air quality regulations	(Ambient air quality Standards Malta, 2010)
Europe	Scotland	3.25	Annual	Objective value	(Air Pollution Information System, 2016)
Europe	Northern Ireland	3.25	Annual	Objective value	(Air Pollution Information System, 2016)
S. America	Colombia	5	Annual	Maximum allowable concentration	(Airlex worldwide Air quality legislation, 2013)
S. America	Peru	4	Annual	Air quality standards	(Decreto Supremo N 074, 2001-Pcm, 2007)
N. America	Cuba	1000	20 min	Maximum allowable concentration	(Airlex worldwide Air quality legislation, 2013)

**Table 3:** Worldwide workplace/indoor standards for benzene.

Name of the Standard	Limit (ppm)	Standard/Definitions	Reference
EH40/2005 Workplace exposure limits	1	Long-term exposure limit (8-hr TWA reference period)	(Health and Safety Executive, 2018)
HKSAR Labour Department	0.5	8-hour occupational exposure limits	(OSH Research Report, 2019)
The National Institute for Occupational Safety and Health (NIOSH)	0.1 1 50 2	NIOSH REL (TWA) - 8-hour occupational exposure limits NIOSH STEL Emergency Exposure Guidance Levels (EEGLs): 1-hour 24-hour	(U.S. Department of health and Human, 2007)
Occupational Safety and Health Administration	1 5	OSHA PEL- 8-hour occupational exposure limits OSHA STEL- 8-hour workday and the maximum short-term exposure limit (STEL) for any 15-minute period.	(OSHA, 1989)
American Conference of Governmental Industrial Hygienists (ACGIH)	0.5 2.5	ACGIH TWA ACGIH STEL	(American Conference of Governmental Industrial Hygienists (ACGIH), 2012)
ANSES**-**French Agency for Food, Environmental and Occupational Health & Safety	20∗ 30∗	Intermediate exposure for 14 days to 1 year. Short-term exposure for 1–14 days.	(Indoor Air Quality Guidelines, 2008)
AIHA-Emergency Response Planning Guidelines	50 ppm 150 ppm 1000 ppm	AIHA ERPG 1 AIHA ERPG 2 AIHA ERPG 2	(AIHA Guideline Foundation, 2016)

∗ - Values are in (μg/m^3^).

## 3 Meta-analysis

**Figure 2:**
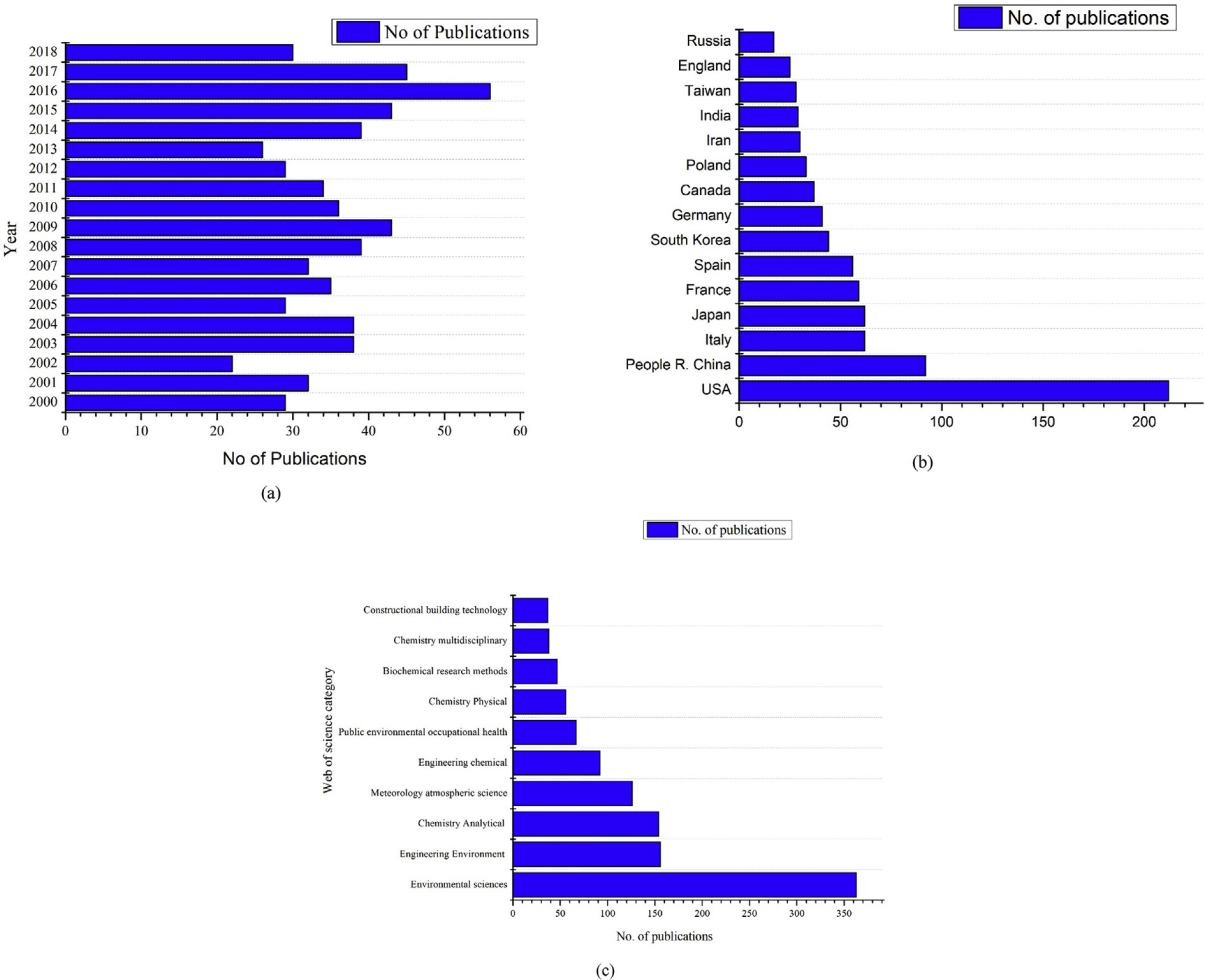
(a) Number of publications vs year (b) Number of publications vs countries (c) Number of publications vs web of the science of category.

From the extensive literature search conducted in the web of science database, a total of 680 papers were obtained on the subject from which information like country of publication (based on the affiliation of the corresponding author), year of publication, web of science category, etc. were extracted. Figure 2a shows the number of publications from the year 2000–2018 on the subject. It is clear from the figure that there is no significant variation in the number of papers published across the years, 2016 being an exception. Thus, though there is an increasing trend in air pollution pertaining to benzene and other VOCs, the number of works conducted has remained almost the same.

Since the subject is part of environmental pollution, the literature obtained was multidisciplinary in nature. Academicians and researchers in the field of environmental science, environmental engineering, applied chemistry, applied physics, biomedical research, instrumentation, etc., are working in the field of monitoring and treatment of benzene emissions. Among all the web of science categories, the top ten categories under which the selected literature on Benzene were published, are listed below in Figure 2c. Other than these listed categories, categories ‘Instrumentation’, ‘Spectroscopy’, ‘Geosciences’ also had a significant number of publications on Benzene. Figure 2b denotes the number of Publications Vs countries/Regions, which indicates that the United States of America produced the highest number of literature when compared with any other country. Though the US and China do not have ambient air quality standard for benzene, these countries produced maximum research literature on the subject.

### 3.1 Sampling and analysis techniques

The most commonly employed sampling methods for Benzene are (i) collection of whole air in special recipients (gas-tight syringes, glass bulbs, plastic bags or metal containers), (ii) collection onto sorbent tubes (Active sampling and passive sampling into sorbent tubes), (iii) continuous sampling and (iv) online sampling (Król et al., 2010; Ras et al., 2009). Most commonly used plastic bags for collection of VOCs are Tedlar, Teflon or aluminized Tedlar bags because of its ease to use, lesser expense and availability in different sizes from 500 mL to 100 L. The main disadvantage of using Tedlar bags for collection of VOCs is that the compounds will not remain stable for more than 24–48 h and there is a high possibility of entrainment of certain chemicals and loss of certain chemicals when the sample is stored for a prolonged period of time (Wang and Austin, 2006). Tedlar bags cannot be used when there is a high difference in relative humidity between the samples and atmosphere. Tedlar bags with double layer are designed with drying agent between two layers to reduce the impact caused by relative humidity (Kumar and Víden, 2007).

Air samples in canisters are collected by free flow at atmospheric pressure (Passive) or with the aid of a pump (active) (McClenny et al., 1991). Canisters are expensive compared to Tedlar bags (Wang and Austin, 2006). Special canisters called summa canisters was designed in the 1970s where the canister is steel coated with a layer of chromium and nickel oxide mixture to reduce reactivity and number of active sites (Hsu et al., 1991). This method is not recommended for benzene and compounds with a large number of carbon atoms because of the wall effect of canisters. According to Compendium of Methods for the Determination of Toxic Organic Compounds in Ambient Air second edition, 1999 released by US-EPA, there are various methods available to monitor benzene based on the variation of sampling methods, pre-concentration, and analytical techniques (US EPA, 1999).

**Figure 3:**
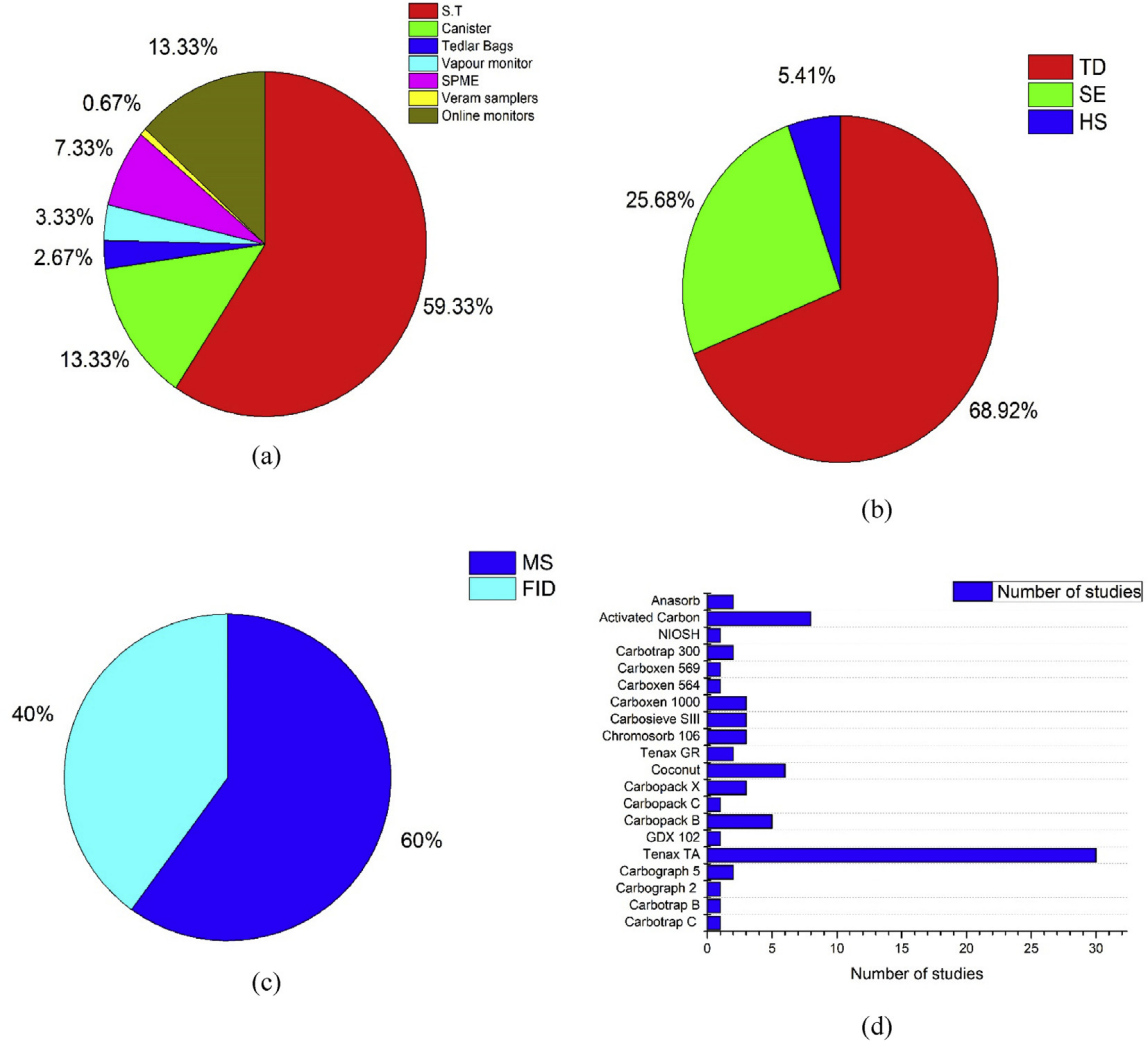
(a) Classification of studies based on sampling method (b) Classification of studies based on sample injection method (c) Classification of studies based on detector (d) Classification based on sorbent material.

In the 151-literature analyzed in details, for about 60% of the studies, sampling was carried out using sorbent tubes. The next common sampling method was using canisters followed by online monitors as in Figure 3a. Samples collected using sorbent tubes can be injected into Gas Chromatograph (GC) either by thermal desorption (TD), solvent extraction (SE) or by headspace (HS) sampling. Thermal desorption was used in 69% of the cases due to its high efficiency which is indicated in Figure 3b. The samples collected from the unpolluted environment cannot be analyzed by solvent extraction because it often requires further addition of pollutants, dilution and re-concentration which may lead to additional error and hence thermal desorption is widely practiced (Harper, 2000). The only major disadvantage of the thermal desorption method of sample injection is the high initial cost. In the solvent extraction method, Carbon disulfide was the most commonly used solvent for extraction due to its good solubilization properties, but it causes serious risk to human health and the environment. Tenax TA filled sorbent tubes were the most commonly employed sorbent tubes for thermal desorption applications (Ras et al., 2009). Among Mass Spectrometry (MS) and Flame Ionization Detector (FID), MS is widely used in the characterization studies which may be due to the presence of the in-built chromatographic library represented in Figure 3c.

## 4 Indoor benzene concentration

### 4.1 Residential

Generally, the levels of benzene in the residential buildings were found to be higher than in the ambient environment in majority of the studies that compared its concentrations in the two environments. The average concentration of benzene found in the living rooms of 26 houses in different types of neighborhoods (urban, suburban and industrial) in Kuwait was 887 μg/m^3^ with the concentrations ranging between 60 μg/m^3^ and 2925 μg/m^3^ (Bouhamra et al., 2000). The mean concentration of benzene, at 15.22 μg/m^3^, exceeded the value recommended by Iran Environmental Protection Agency in the selected 50 residential homes of Ardabil city, Iran. Among the BTEX compounds, the hazard quotient for benzene exceeded the acceptable level and the indoor benzene concentrations were found to be contributed by the heating system, story, and the usage of natural gases (Hazrati et al., 2016a, Hazrati et al., 2016b). Effect of ventilation on indoor VOCs was studied in apartments built according to the South Korean Clean-Healthy House construction standard during December 2010. The level of benzene was 1.3 μg/m^3^ without ventilation and 0.6 μg/m^3^ with ventilation (Kim et al., 2017). In Shanghai, China, 20 homes of asthmatic children were selected to measure exposure to toxic VOCs during the sleeping hours. The sampling was carried out for two conditions. In one case, the room was fitted with an air purifier where the filter contained three layers of filtration i.e., washable pre-filter to capture large particles, replaceable High Efficiency Particulate Air (HEPA) filter to remove smaller particles and a replaceable activate carbon filter to remove odour. In the second case the room was fitted with a sham air cleaner, where the filter does not contain HEPA and activated carbon filter. The mean concentrations of benzene were 2.6 and 3.1 μg/m^3^ with true and sham filtration, respectively. They have also found that benzene contributes to 13% of the total inhalation cancer risk (Fang et al., 2019). Results showed that air purifier may reduce concentration of a few VOCs in the indoor environment. In a similar study conducted in sub-urban homes of Shanghai, VOCs were measured to evaluate the impact of air filtration. The average benzene levels found in 20 homes were 2.9 μg/m^3^ and 2.6 μg/m^3^ with sham and true air filtration, respectively. The mean outdoor concentration was 3.1 μg/m^3^ in both these cases (Norris et al., 2019).

According to a report published by world energy outlook in the year 2016, around 819 million people, i.e., 62% of the total population use traditional biomass cookstoves for cooking in India and the emissions from this cook stove leads to household air pollution which in turn causes premature death across the country every year (Council of Energy Environment and water, 2017). A study conducted by the National Institute of Occupational Health (ICMR), India showed that the level of benzene in kitchens using dung fuel was 114.1 μg/m^3^, wood fuel-36.5 μg/m^3^ and open type kitchen 6.6 μg/m^3^. The sampling, in this case, was carried out using a personal sampler to collect samples from both indoor and open type kitchens. The sampler was placed such that it was in the breathing zone of persons and 30 cm away from cooking activity (Sinha et al., 2005). In a similar manner, studies on benzene exposure to cooks during cooking using mixed fuel, i.e., a combination of cow dung and wood, were carried out in villages where the people reside in homes with one room or 2 to 3 rooms with partitions, indoor kitchens, open type kitchen, ventilated and non-ventilated kitchens. The level of benzene was found to be 75.3 μg/m^3^ inside kitchens with a partition wall, 63.206 μg/m^3^ without partition, 11.7 μg/m^3^ in open type, 31.2 μg/m^3^ in ventilated and 45 μg/m^3^ in non-ventilated. Benzene produced while burning cow dung was significantly higher when compared with wood and the cooks in open type kitchens experienced less exposure to benzene (Sinha et al., 2006). The average level of benzene found in a residential building in Kolkata, India was 42 μg/m^3^, which is eight folds higher than the National Ambient Air Quality Standard (NAAQS) of the country. The sampling was carried out in 45 residences distributed in three different communities. The sampler was placed 1 m above the floor level and 1–2 m away from any walls and obstructions. The study showed that fuel type and ventilation were the factors influencing indoor benzene concentrations. The location of the building and the position of the kitchen inside the building had no influence (Majumdar et al., 2012).

The concentrations of benzene reported from European homes are significantly less than those from Indian homes, but, the values exceed the countries’ standards in many cases. A study carried out in Greece covering 25 homes, with sampler kept 1.5 m above the floor level in the middle of the sitting room, showed benzene levels of 15.3 ± 8.0 μg/m^3^. The mean concentration of benzene was higher during the winter season and the level of benzene found in smokers home was higher than the level found in non-smokers home (Baya et al., 2004). A similar study was conducted in Gothenburg, Sweden, where the highest level was found to be 1.6 μg/m^3^ (non-smoker home) and 3.8 μg/m^3^ (smokers home) (Strandberg et al., 2006). In a study carried out in homes with children up to 1-year age, in the city of Valencia, Spain, and 34 of surrounding villages and medium-sized towns, which included 352 houses, the average concentration of benzene found inside the houses (2.7 μg/m^3^) was 2.5 times higher than the outdoor benzene level (1.2 μg/m^3^). A few of the other interesting results obtained from this study were; the level of benzene was higher in the house with mothers who are of non-Spanish origin, benzene level was lesser in air-conditioned houses and the level of benzene was higher in houses which uses electric cooking rather than in houses which use cooking gases (Esplugues et al., 2010). Indoor benzene sampling was carried out in the homes of the elderly at Antwerp city center and several suburban areas like Broechem, Borsbeek Hove and Bonheiden located in Belgium and the level exceeded the permissible Flemish standard (2 μg/m^3^) in all sampling location at Broechem (2.5 ± 2.9 μg/m^3^) (Walgraeve et al., 2011). Indoor air samples from the living rooms and bedrooms of 777 homes in Hamburg and three other regions of East Germany were analyzed for benzene. The sampling was performed twice at an interval of 7 months and the median level was found to be 2.5 μg/m^3^ (Living room) and 2.1 μg/m^3^ (Bedroom). They found that there was a very crude correlation between the readings taken during the two visits (Rebekka Topp et al., 2004).

In an extensive study carried out in Canadian residential buildings, which included 3218 houses, 546 apartments, and 93 other dwelling types, covering five different regions (Atlantic, Quebec, Ontario, Prairies and British Columbia), the average level of benzene was found to be 1.93 μg/m^3^ (Zhu et al., 2013). In another study conducted in a residential building and a chemical store in the city of Waterloo, Ontario, Canada, the average concentrations of benzene were found to be 248 ppb (garage), 1.3 ppb (main office) and 14.8 ppb (waste receiving room) (Jia et al., 2000). In a study conducted in Alaska, samples were collected from the living room of two residential buildings and the concentration of benzene was between 1-25 ppbv. It was concluded that gasoline stored in garages outside the houses was the major contributor of benzene and other aromatic organic vapors (Isbell et al., 2005). In southeast Chicago, sampling was carried out in 10 urban homes from highly industrialized region, which included five homes from Altgeld Garden area, two homes in the Torrence Avenue area, two homes in the Beverly area and one home in Calumet City, and the average level of benzene was found to be 4100 ng/m^3^. It was found that indoor VOC concentrations and emission were influenced by product use and occupant activities (Michael R. Van Winkle & Peter A. Scheff, 2001). In a study conducted in residential homes of Detroit, Michigan, the concentration of benzene was found to be 3 ± 5.7 μg/m^3^, and the indoor to the outdoor ratio for benzene was 1.2 (Johnson et al., 2010). The level of benzene in a newly built home was measured using adsorption/combustion-type gas sensor and was found to be 3 μg/m^3^. The results obtained agreed well with the results obtained in GC-MS and GC-FID (Sasahara et al., 2007). Between December 2003 and April 2006, the level of benzene found in one hundred rural and urban homes from 13 counties across the State of New-Jersey was 4.07 ± 5.94 μg/m^3^ (Weisel et al., 2008).

### 4.2 Commercial

Several studies have proved the emission of VOCs from health and personal care products. The average level of benzene found in fifty beauty salons in Ardabil, Iran was 32.40 ± 26.38 μg/m^3^ and the study showed that the level of benzene was influenced by ventilation, number of occupants, area of the room and the number of services (Norouzian et al., 2018). Similarly, in Ardabil City, Iran the mean concentration of benzene found in 81 waterpipe cafés was 4.96 ± 2.63 mg/m^3^. It was found that flavored tobacco contributes more BTEX when compared with regular tobacco (Hazrati et al., 2015). The level of benzene measured in the indoor air of a dental hospital in Italy (4.4 μg/m^3^) was higher when compared with the outdoor air (0.5 μg/m^3^) (Santarsiero et al., 2009). In Kuopio University Hospital, Finland, floor materials were monitored for VOC emission to know their contribution to the indoor VOC levels. It was found that the mean concentration of benzene emitted from the floor materials was 0.8 μg/m^3^ and its concentration in the indoor air was 1.2 μg/m^3^. But, the linear regression model used in the study, did not confirm the influence of floor emissions on indoor VOCs (Rautiainen et al., 2018). The concentration of benzene in a multistory shopping mall in the suburbs of Bari, Italy was found to be 0.10–5.28 μg/m^3^ and 0.60–9.14 μg/m^3^ in the first and second sampling campaign, respectively, carried out at a gap of 10 months. The average Indoor/Outdoor (I/O) ratio of benzene in supermarket and storehouses were 0.8 and 0.9 respectively which according to the authors indicated that the benzene in the indoor environment is due to the outdoor benzene (Amodio et al., 2014). The level of benzene found in the gasoline shops at the City of Belo Horizonte, Brazil was 39.81 ± 63.30 μg/m^3^ (Helvécio C. Menezes et al., 2009).

The concentration of benzene in a car body shop in Italy was found to be 0.2 ppm which was twice the permissible level (Mastrogiacorno et al., 2000). The concentrations of benzene measured at a few commercial establishments were; paint shop - 15 ppb, grinding shop - 0.90 ppb, carpenter shop - 10 ppb (Koziel and Pawliszyn, 2001), food courts - 7.44 μg/m^3^, theater - 30.95 μg/m^3^, bar floor - 27.18 μg/m^3^ and restaurants - 2.58 μg/m^3^ (Srivastava and Devotta, 2007). The average level of benzene found in the smoking areas of ten restaurants in Helsinki was 3.6 μg/m^3^ (Vainiotalo et al., 2008). Sampling was done at ten points within the city of Chuncheon, Korea where at least three charcoal restaurants functioned nearby, and found the average benzene level to be 2.93 ± 1.41 μg/m^3^, which was 1.3–2.6 folds higher than the level found in non-charcoal restaurants areas (Kim and Lee, 2012).

Several researchers have attempted to study the level of benzene in the interiors of vehicles. Some of the observations were; 32 ± 3 μg/m^3^ (Maximum) inside various motor vehicles for a long exposure period of 24 h (Esteve-turrillas et al., 2007), 13.8 μg/m^3^ inside passenger cars running in gasoline without air freshener, 14.3 μg/m^3^with air freshener - 8.7 μg/m^3^ inside diesel cars without air freshener and 8.3 μg/m^3^ with air freshener (Jo et al., 2008) and 21.3–106.4 μg/m^3^ inside public buses in China (Chen et al., 2011).

### 4.3 Industrial buildings

The level of benzene found in an Liquid Crystal Display fabrication center was 1.5 ppb (Wu et al., 2004). The benzene concentrations at the two boiling water reactors in a power plant in Taiwan were 3.25 ± 0.94 ppb and 1.31 ± 0.04 ppb (Hsieh et al., 2006). Personnel exposures to benzene for the occupants of a petrochemical industry were found to be 27.80, 40.00 and 24.20 μg/m^3^ in case of Petrochemical industry operators, Service station attendants and gasoline pump maintenance workers, respectively (Enrica et al., 2010).

### 4.4 Institutional buildings

Higher levels of benzene were observed in the educational and research institutions in different parts of the world. Inside an organic chemical laboratory, it was found to be 0.30 ppb (Lee et al., 2002). 1 μg/m^3^ of benzene was observed in classrooms at the Aquitaine region near Bordeaux, France (Larroque et al., 2006). The benzene concentrations observed in the conference room and office room of a university building in Mumbai, India was 113.89 μg/m^3^ and 0.8 μg/m^3^, respectively (Srivastava and Devotta, 2007). Benzene in the laboratory air measured at the city of Belo Horizonte, Brazil was 3.41 ± 1.98 μg/m^3^ (Helvécio C. Menezes et al., 2009). 173 office buildings in southern Finland had concentrations in the range of 0.2–4 μg/m^3^ (Salonen et al., 2009). The 95^th^ percentile indoor benzene level in the primary schools at I˙zmir, Turkey was 29 μg/m^3^ and benzene was the third most abundant compound next to formaldehyde and toluene (Sofuoglu et al., 2011). In the south-central part of Spain sampling was conducted in kindergartens located in rural, urban and industrial regions and the concentrations of benzene were, 0.3 ± 0.1 μg/m^3^, 0.5 ± 0.1 μg/m^3^, 0.9 ± 0.6 μg/m^3^, respectively, during February to April 2013. It was concluded that the higher concentration of benzene in the kindergarten located in the industrial region is due to the contribution from the petrochemical plant (Villanueva et al., 2018). In naturally ventilated school buildings at Taranto City, South of Italy, three classrooms were selected for benzene monitoring and the average concentration found in all the three were 0.44 μg/m^3^ which was less than the outdoor ambient benzene levels (0.67 μg/m^3^) (Marzocca et al., 2017). In Czech technical university in Prague, the average level of benzene in indoor air was 1.6–4.9 μg/m^3^ during June 2012 (Kolarik et al., 2015). In a study conducted at two different schools in Osijek, Croatia the levels of benzene in the indoor air were found as 0.44 μg/m^3^ and 1.63 μg/m^3^, whereas the ambient benzene level was 0.65 μg/m^3^ in both the cases. The higher concentration in the second school was attributed to the new floor lamination and the position of the windows, which were near to possible benzene sources (Brdarić et al., 2019).

## 5 Outdoor benzene concentration

### 5.1 Urban and roadside location

#### 5.1.1 Asian countries

The concentration of benzene found in the ambient air of Kula Lumpur, Malaysia was 18.2 ± 12.9 ppb during December 2013–January 2014 (Hosaini et al., 2017). In the roadside areas of Changchun, northeast of China, the average level of benzene found during the period from September 1997 to July 1998 was 38.5 μg/m^3^ (Liu et al., 2000). In Northeastern urban region of Beijing, China the mean concentration of benzene found from August 24 to September 4, 2012 was 11.98 μg/m^3^ (Li et al., 2014). In the year 2001, the levels of benzene were measured before the typhoon, during a typhoon and after typhoon *Nari* and the concentrations were 1.40, 0.27 and 0.71 ppb, respectively, in and around Hsinchu Science Park, Taiwan (Nian et al., 2008). In China's most developed coastal regions of Shenyang, Yucheng, Taihu and Dinghu the concentrations were found to be in the range 578–1297 ppt during the sampling period, from March 2012 to February 2013 (Zhang et al., 2015). At urban road-side locations of Hong-Kong China, the concentrations found in the year 2003 and 2011 were 1408 and 906 ppt, respectively, and the benzene pollution was found to be decreasing with year (Huang et al., 2015). In the southernmost part of Taiwan during 2010, the level of benzene found in urban and beach locations were 0.80 and 0.32 ppb respectively (Liu et al., 2012).

In Japan, the level of benzene found at Chiba city in February and March 1999 was in the range of 2–10 μg/m^3^ and 1.5–8.6 μg/m^3^, respectively (Uchiyama and Hasegawa, 2000). In Tokyo urban sites, the level of benzene found during the sampling years 2003 and 2004 were 2.5 ± 1.2 μg/m^3^ and 4.0 ± 1.8 μg/m^3^, respectively (Hoshi et al., 2008). Studies were carried out in the outdoor parking lots in Yokohama and Kawagoe, Japan and the benzene level was found to be 82.7 μg/m^3^ (Tokumura et al., 2016). In rural locations of Tokyo, Japan the level of benzene found in the year 2003 and 2004 were 4.6 ± 1.6 μg/m^3^ and 2.3 ± 1.1 μg/m^3^, respectively. The level in rural regions was higher when compared with urban locations in the year 2003 and it was vice versa in 2004 (Hoshi et al., 2008). Studies carried out in Bangkok, capital of Thailand, also showed very high concentrations of benzene; 15.1–42.4 μg/m^3^ in the peak hours and 16.3–30.9 μg/m^3^ in the non-peak hours, during January to December 2000 (Tet et al., 2002). Studies during the rainy and summer season of 2012 and 2013 showed that the concentrations were still high, with mean value at 45.5 μg/m^3^ (Kanjanasiranont et al., 2015).In the capital city of South Korea, the benzene concentration was monitored in four different locations during February to December 2009 and the range was found to be 0.56 ± 0.34 ppb (Kim et al., 2012).

In Kazakhstan located in central Asia, sampling was carried out in five different districts and the mean concentration was found to be 53 μg/m^3^ during 2015 (Baimatova et al., 2016). In Tehran, the capital city of Iran, the average concentration of benzene found in the year 1996–1997 was 127.6 μg/m^3^ in the regions where the traffic flow was in the range of 500–2500 vehicles/hr (Bahrami, 2001). In another study at Tehran, carried out from 5 April 2010 to 25 March, 2011 the benzene concentration was 14.51 ± 3.17 ppb in the traffic zones (Atabi et al., 2013). Similarly, in Ahvaz metropolitan city, from January to March 2013, the level of benzene was found to be 1.78 μg/m^3^ (Rad et al., 2014).

Turkey is a country straddled between eastern Europe and western Asia. Benzene measurements at Izmir, Turkey showed concentrations of 11.6 ± 3.2 ppb in the month of August and 17.5 ± 2.6 ppb in the month of September 1998 (Muezzinoglu et al., 2001). In a study conducted at Kaynaklar campus of the DokuzEylul University (Sub-urban), Izmir, Turkey which is located 10 km southeast of Izmir's city center, abutting streets with heavy traffic (urban site), the average concentrations were found to be 0.85 ± 0.40 μg/m^3^ (Summer) and 10.40 ± 8.96 μg/m^3^ (winter), respectively (Elbir et al., 2007). In Dhaka, the capital of Bangladesh, the level of benzene was found to be very high. Concentrations up to 10560 μg/m^3^ were noticed in public places here (Hussam et al., 2002). In the capital of Vietnam, studies were carried out in different roadside locations and the concentrations were found to be ranging from 65 μg/m^3^ to 123 μg/m^3^ (Truc and Oanh, 2007). The amount of benzene, measured using PTR-TOF-MS, in the Kathmandu valley in Nepal during the winter of 2012–2013 was 2.72 ppb (Sarkar et al., 2016).

Many major Indian cities like Delhi, Mumbai, Kolkata are among the most polluted cities of the world (WHO, 2018). But very few studies have been conducted in these places to record the concentration of benzene and the ones carried out have shown very high concentrations of benzene in the ambient air. The maximum level of benzene found in the ambient air of some locations in Mumbai were; Haji Ali crossing - 573 μg/m^3^, Worli crossing - 886 μg/m^3^, Kalbadevi crossing - 1545 μg/m^3^, Dadar arterial road - 781 μg/m^3^, Mahim arterial road - 282 μg/m^3^, Western express highway - 960 μg/m^3^ and Eastern express highway - 246 μg/m^3^ (Srivastava and Singh, 2005). The level of benzene in Delhi was found to be; 33.69–174.62 μg/m^3^ in Preet Vihar, 13.03–33.06 μg/m^3^ in ITO and 17.69 μg/m^3^ in East Arjun Nagar (Kumar and Tyagi, 2006). To reduce air pollution, the Government of Delhi made it mandatory to use Compressed Natural Gas (CNG) for public transportation including taxis. Benzene was monitored both in the pre-CNG (October 2001 to September 2002) and post-CNG period (January to February 2007) and the concentrations were found to be 116.32 ± 51.65 μg/m^3^ and 187.49 ± 22.50 μg/m^3^, respectively during winter. The increase in benzene concentration in spite of shifting to CNG was attributed to the very high increase in the vehicle population, from 3.5 million (in 2001) to 5.1 million (in 2007) (Khillare et al., 2008). In another study carried out in Delhi on the emissions and air-quality during the fog events during 2015–16 and 2016–17, it was found that the average benzene concentration during fog events was approximately 5.6 ppb. During the winters of 2015–2017 the highest level of benzene was found to be 20 ppb. Considering at least 48 days of fog events every year with an average benzene concentration of 5.6 ppb, the population inhales approximately 269 ppb in a short span of time. This amounts to 46% of permissible annual intake limit. The benzene was found to be sourced both from biomass burning and vehicular emissions (Hakkim et al., 2019). In a study conducted in Gorakhpur, the Terai zone of Northern India, the average benzene concentration measured in the roadside areas, was 15.9 μg/m^3^ (Masih et al., 2016).

#### 5.1.2 European countries

The average level of benzene observed along the road margins at selected places in Belgium, Hungary and Latvia were 3.07, 2.30 and 7.80 μg/m^3^, respectively in the year 1995. The concentration measured in Latvia exceeded the limit prescribed by the European Union (Regine et al., 2001). The concentration of benzene measured in the urban air of La Coruña, Spain in the year 1996 was 9.48 ± 20.83 μg/m^3^ (G. Fernández-Martínez et al., 2001). Similarly, in A Coruña (NW Spain), the level of benzene was 3.43 ± 4.08 μg/m^3^ in the Winter of 2000 (V. Ferna'ndez-Villarrenaga et al., 2004). In the same street canyon of A Coruña (NW Spain), a study was carried out during November–December 2000, and the average level of benzene was found to be 2.69 ± 2.26 μg/m^3^ (Fernandez-villarrenaga et al., 2005). 27.9 μg/m^3^ (maximum) was the concentration measured in the urban air of Tarragona, Spain in December 2005 (Ras-Mallorquí et al., 2007). In Cabauw, The Netherlands, a quasi-urban site close to major Dutch cities, the average level of benzene observed was 440 ppt (Warneke et al., 2001). In the north-eastern part of Italy, the level of benzene was found to be 2.3 μg/m^3^ in the sub-urban region having low traffic intensity and 10.3 μg/m^3^ in the area having high traffic during the sampling period from June 1998 to May 1999 (Bono et al., 2003). In the urban area of Verona, Italy, the annual average of benzene found in the year 2012 was 1.81 μg/m^3^ (Schiavon et al., 2015). In the urban air of Rome, sampling was carried out in four different seasons during 2011 and the concentrations were found to be 3.29 ± 1.61, 2.04 ± 0.82,1.53 ± 0.55, 2.01 ± 1.19 μg/m^3^ in winter, spring, summer and fall, respectively (Fanizza et al., 2014). In Torino and Pragelato, the concentration found during May 2005–April 2006 was 3.2 ± 2.4 and 1.7 ± 1.4, μg/m^3^ respectively (Bono et al., 2010). In Padua, sampling was done in two different locations and the concentrations found were 7.39 (max) and 10.12 (max) μg/m^3^ (Sturaro et al., 2010). The concentration of benzene found in the urban area near to road traffic tunnel portals of Naples was 23.5 μg/m^3^ (max) at Via Caio Duilio and 21.6 μg/m^3^ (max) at Via Fuorigrotta during May to October of 2004 (Murena, 2007). In Lille, a city in France, the level was found to be 1.5 μg/m^3^ (median) during April 1997–May 1999 and 0.61 μg/m^3^ (median) during July 1999–June 2000 (Borbon et al., 2004). In an industrialized harbor of Northern France, 8 h sampling was performed for benzene during a sea breeze event (July 16th, 2009) and the level was found to be 4.9 ± 0.3 μg/m^3^ (Roukos et al., 2011). The benzene levels in Douai, Dunkerque, and Marseille, measured at different time periods between 2003 and 2004, were in the range of 0.5–2.6 μg/m^3^ (Buzica et al., 2008). The concentration of benzene found in the North-east coast of Sweden during Autumn 2001 was in the range of 0.7–17 μg/m^3^ (Modig et al., 2004). Similarly, during Autumn 2000 to 2001 in the State of Schleswig-Holstein, Germany, the average concentration of benzene was 2.9 μg/m^3^ (Hippelein, 2004). In Greece, the concentration of benzene found in the Urban city center was 0.93 ± 0.55 ppb during 2003 and 2004 (Kelessis et al., 2006). A number of studies have been conducted in Poland especially in Gdansk. In Zabrze, Poland the level was 8.1 μg/m^3^ during August–September 2001 and 2.8 μg/m^3^ during August–September 2005 (Pyta, 2006). In a study conducted in 2012, the levels of benzene found in Gdansk, Gdynia and Sopot were 0.72 ± 0.11, 0.66 ± 0.51 and 0.63 ± 0.55 μg/m^3^, respectively (Marć et al., 2014a). In Gdansk it was found to be 0.66 ± 0.32 μg/m^3^ during January to December 2013 (Marć et al., 2015) and 0.14–2.37 μg/m^3^ in Gdansk and Gdynia during March and December 2011 (Marć et al., 2014a, Marć et al., 2014b). Benzene concentrations were measured at a few locations in Nisyros Island and the concentration were found to be 5.4, 22, 0.5 and 0.19 μg/m^3^ in Lakki plain, Mandraki village, northern seashore and the Lakki Caldera rim, respectively (Tassi et al., 2013).

#### 5.1.3 American countries

The number of studies conducted in the United States of America is significantly higher when compared with studies conducted in other parts of the world. The level of benzene found in ambient air near to four schools and avenues of Southeast Chicago during June 1994–April 1995 was 1,246 ng/m^3^ (Michael R. Van Winkle & Peter A. Scheff, 2001). The average level of benzene measured at ‘State of Texas Commission on Environmental Quality Continuous Air Monitoring Station’ from November to December 1999 was 9.19 ppb (Mukerjee et al., 2004). In a study conducted in the ambient air of 13 semi-rural to urban locations in the United States during 1998–2000, the mean concentration of benzene was 1.07 ppb (Pankow et al., 2003). Similarly, the level of benzene in the ambient air was 0.1 ± 0.2 ppb, and in the roadside region of major traffic routes, 0.2 ± 0.3 ppb, in a study conducted in the rural and urban region of Wake County (Riediker et al., 2003). In Pittsburg, the median value of benzene level found during July 2001 through August 2002 was 279 ppt during winter and 215 ppt during summer. In South Camden part of New Jersey, the level of benzene found in the village of Waterfront South neighborhood, a “hot spot” for air toxics in Camden, was 1.2 ± 1.3 μg/m^3^ and in Copewood/Davis Streets neighborhood, an urban reference area located ~1000 m east of the Waterfront South, it was 1.4 ± 1.2 μg/m^3^ during 2005 (Zhu et al., 2008). Benzene level detected in Lynchburg, measured using Automatic-GC, was 912.74 ppb (maximum value) (Raun et al., 2009). In south coast air basin of California, monitoring was carried out at Photochemical Assessment Monitoring Stations from 1999 to 2009 and the mean benzene concentration was found to be 0.4 μg/m^3^ (Pang et al., 2015). In urban and rural locations of New-York state, sampling was carried out between 1990-2003 and the mean concentration of benzene were, Buffalo (Industrial) - 5.09 μg/m^3^, Brooklyn (urban) - 2.85 μg/m^3^, Hudson Valley (small urban) - 2.31 μg/m^3^, Niagara Falls (Urban Industrial) - 1.80 μg/m^3^ and Adirondacks (Rural) - 0.86 μg/m^3^ (Aleksic et al., 2005). The mean concentration of benzene found in Pittsburgh (6 km east of downtown Pittsburgh) was 279 ppt and 215 ppt during winter and summer season respectively (Millet et al., 2005). In Deer Park, Texas near Houston VOCs were monitored in 12 different sites within 3 km radius during summer 2003 and the average level of benzene was 2.04 μg/m^3^ (Smith et al., 2007). Air quality Monitoring was carried out in two sites operated by the State of Michigan Department of Environmental Quality at East 7 Mile and Dearborn and the median level was found to be 2.6 ppb and 2.2 ppb respectively during summer 2005 (Mukerjee et al., 2009).

In Nuevo Leon, Mexico, during Summer and Autumn 2013, the average concentration of benzene was found to be 55.24 μg/m^3^ (Cerón-Bretón et al., 2015). The level of benzene found in the avenues at the City of Belo Horizonte, Brazil, was 18.26 ± 17.9 μg/m^3^ (Helvécio C. Menezes et al., 2009). The mean concentration of benzene found in the Metropolitan Region of Sao Paulo, Brazil was 2.6 ppb during 1998, where all the samples were collected during busy traffic hours (Colón et al., 2001). In La Plata and neighboring areas, located 50 km south-eastern of Buenos Aires (Argentine) the mean concentration of benzene found during one winter month sampling for three consecutive years from 2000 and 2002 were; industrial region - 16.10 μg/m^3^, urban region - 3.15 μg/m^3^, semi-rural region - 1.64 μg/m^3^ and the median level of benzene in residential region - 1.52 μg/m^3^ (Massolo et al., 2009).

Benzene monitoring was carried out in Canada to know the effectiveness of the implementation of the Canada-wide Standard for Benzene by the Canadian Council of Ministers of the Environment. The standard, implemented in two phases had targeted a 30% reduction in benzene emissions in phase 1 by the year 2000, taking 1995 as the base year. In phase 2, the target was a further 12% reduction by the year 2010. Monitoring was carried out in 10 urban locations and 6 rural locations. In the urban locations, the annual average ambient concentrations decreased to 0.93 μg/m^3^ in 2009 from 3.60 μg/m^3^ in 1994 and in rural locations, the level of benzene remained stable. This reduction was attributed to the introduction of the federal ‘Benzene in Gasoline Regulations’ and due to best management practices by the Canadian Association of Petroleum Producers (Canadian Council of Ministers of Environment, 2012).

#### 5.1.4 Australian and African countries

Only very few published works were noticed from Australian and African countries during the literature search. The level of benzene measured at Cape Grim, Tasmania was 4 ± 1 ppt during a monitoring campaign from 10^th^ February to 1^st^ March 2006, intended to measure the concentration of compounds in the unpolluted ocean air. This concentration was much less than the corresponding concentration in the air of northern hemisphere tropical ocean (53 ppt) (Galbally et al., 2007). In Cairo, the capital city of Egypt, the level of benzene was monitored in seven different sites during winter 1999 and the highest of the average among the seven was 43 ± 7 ppb (Abu-Allaban et al., 2009).

### 5.2 Industrial and agricultural sites

The outdoor air at a location having many chemical industries in the South-East of France was monitored for benzene and the concentration observed was 4.0 μg/m^3^ (Tumbiolo et al., 2004). The level of benzene found in the urban air near a landfill facility in South Korea during the winter of 2004 was 0.99 ± 1.10 ppb (Kim et al., 2008). The benzene concentration in Gorakhpur Industrial Development Area of Terai zone, North India sampled during Nov. 2014 to Oct. 2015 was found to be 29.2 μg/m^3^, which was higher compared with the samples taken in residential, roadside and agricultural locations during the same study. The concentration of benzene found in the residential regions of Terai zone in Northern India was 7.1 μg/m^3^. The concentration of benzene found in the outdoor air of an agricultural location in Haiderganj, Uttar Pradesh was 11.4 μg/m^3^ (Masih et al., 2016). In the industrial region of Tarragona, Spain, the highest concentration of benzene found in the outdoor air was 16.1 μg/m^3^. The sampling was done during December 2005 and January 2006 (Ras-Mallorquí et al., 2007). The concentration of benzene found in Galena Park, Texas near the Houston Ship Channel was 37 ppb (maximum value) (Olaguer et al., 2015). The concentration of benzene found in the air at a winery close to Valencia (Spain) was 424 - ng/m^3^ (Sanjuán-herráez et al., 2014). Average benzene found within the degassing carters in Nisyros Island was 23.75 μg/m^3^ during September 2010 and 2012 (Tassi et al., 2013). Samples were collected from two different sites near an Industrial WWTP located in the South Industrial Complex, Tarragona area of Spain, during November 2008 and the levels of benzene were 1.31 and 1.46 μg/m^3^ (Ramírez et al., 2011). Air samples were collected around the different industries in Daegu, Korea during June–September 2004 and that the benzene concentrations were; around wastewater incinerator- 218.1 ± 346.5 ppb, food manufacturing industry - 5.2 ± 0.9 ppb, chemical manufacturing industry 10.6 ± 7.9 ppb, excretion disposal facilities - 4.6 ± 0.3 ppb, sewage & wastewater processing facilities 4.5 ± 0.0 ppb, general garbage incinerator - 3.6 ± 0.0 ppb, fiber manufacturing industry - 6.8 ± 2.4 ppb, paper manufacturing industry - 4.8 ± 0.1 ppb and food manufacturing industry with alcohol - 4.6 ± 0.3 ppb (Choi et al., 2009). The level of benzene was continuously monitored around the boundary of a coking plant in the United Kingdom, and was 28 μg/m^3^, between 2004 and 2006. The measurements were made using Differential Optical Absorption Spectrometry (Ciaparra et al., 2009).

### 5.3 Petrol stations

Petrol and diesel stations are among the most important sources for benzene in the ambient air. In a study conducted in the city of Murcia, Spain, the spatial influence of pollutants from petrol station was determined by establishing a ratio between aliphatic and aromatic pollutants in the air of petrol stations and the surrounding areas. Benzene, n-hexane and cyclo-hexane were the VOCs selected for the study. The average concentration of benzene found in the petrol station during the first sampling campaign was 8.98 μg/m^3^ and 18.4 μg/m^3^ during second sampling campaign. From the results obtained it was found that the influence of the petrol station was higher within 75 m of its boundary (Morales Terrés et al., 2010). From a similar study conducted in the urban, sub-urban and rural regions of Greece, it was concluded that there is a clear influence of petrol stations in the surrounding benzene concentrations, for all the three locations. It was also found that the people living in the vicinity of the petrol stations were exposed to 6–9 μg/m^3^ of benzene, at least for 10 h/day, which increased their cancer risk from 3% to 21% depending on the exposure times (Morales Terrés et al., 2010). The level of benzene found in and around Chinese petroleum corporation (CPC) refinery at Kaohsiung, located in southern Taiwan was 618 ppb (max) in the morning and 64.3 ppb in the afternoon, when monitored from 28 April to 4 May 2001 using Gas chromatography and ultra-violet differential optical absorption spectroscopy (UV-DOAS) (Lin et al., 2004). Studies were carried out to estimate the benzene released during refueling of gasoline and diesel gasoline tanks. It was found that 330 ± 9, 4900 ± 200, 1670 ± 40 μg/m^3^ of benzene was emitted while filling 5L, 10 L and 20 L gasoline tanks, respectively and 1900 ± 40, 2320 ± 80, 1100 ± 100 μg/m^3^ while filling 5L, 10 L and 20 L diesel tanks, respectively (Esteve-turrillas et al., 2007). Similar studies have been carried out in ambient air of refueling stations in Ardabil city and the concentration in the air was found to be 2.01 mg/m^3^ (Hazrati et al., 2016b). Two types of measurement were carried out in the Barnett Shale in north-central Texas, one of the largest, most active onshore gas fields in North America. One was using Auto-GC and the other by collecting samples using canisters followed by analysis in GC-MS. It was found that the values obtained were close, with Auto-GC giving a concentration of 0.528 μg/m^3^ and the second method, 0.664 μg/m^3^ (Bunch et al., 2014). In Tehran, Iraq, the average benzene concentration found in ambient air near to four gas stations was 29.01 ± 1.322 ppb after monitoring the air for one year (Atabi et al., 2013). To access the impact of shale gas operation at Barnett Shale region in the North-central Texason's VOCs concentration, monitoring was performed in seven monitoring stations and the annual average benzene concentration at all the monitoring station ranged from 0.341 μg/m^3^ to 0.815 μg/m^3^ (Bunch et al., 2014).

## 6 Summary of benzene monitoring studies

Children and elderly people spend most of their time in residential environments. Personal and home care products, newly bought furniture, carpets, paints and activities like cooking contribute to indoor VOCs. In residential environments, a comparison was made among houses in Asian, European and North American countries for benzene levels. The average level was found to be 111 μg/m^3^ in Asian countries, wherein the studies conducted in India had an average of 50 μg/m^3^. The higher concentrations were reported from homes where biomass fuel is used for cooking. The average level of benzene found in European and North American countries were 4.7 and 6.3 μg/m^3^, respectively.

**Figure 4:**
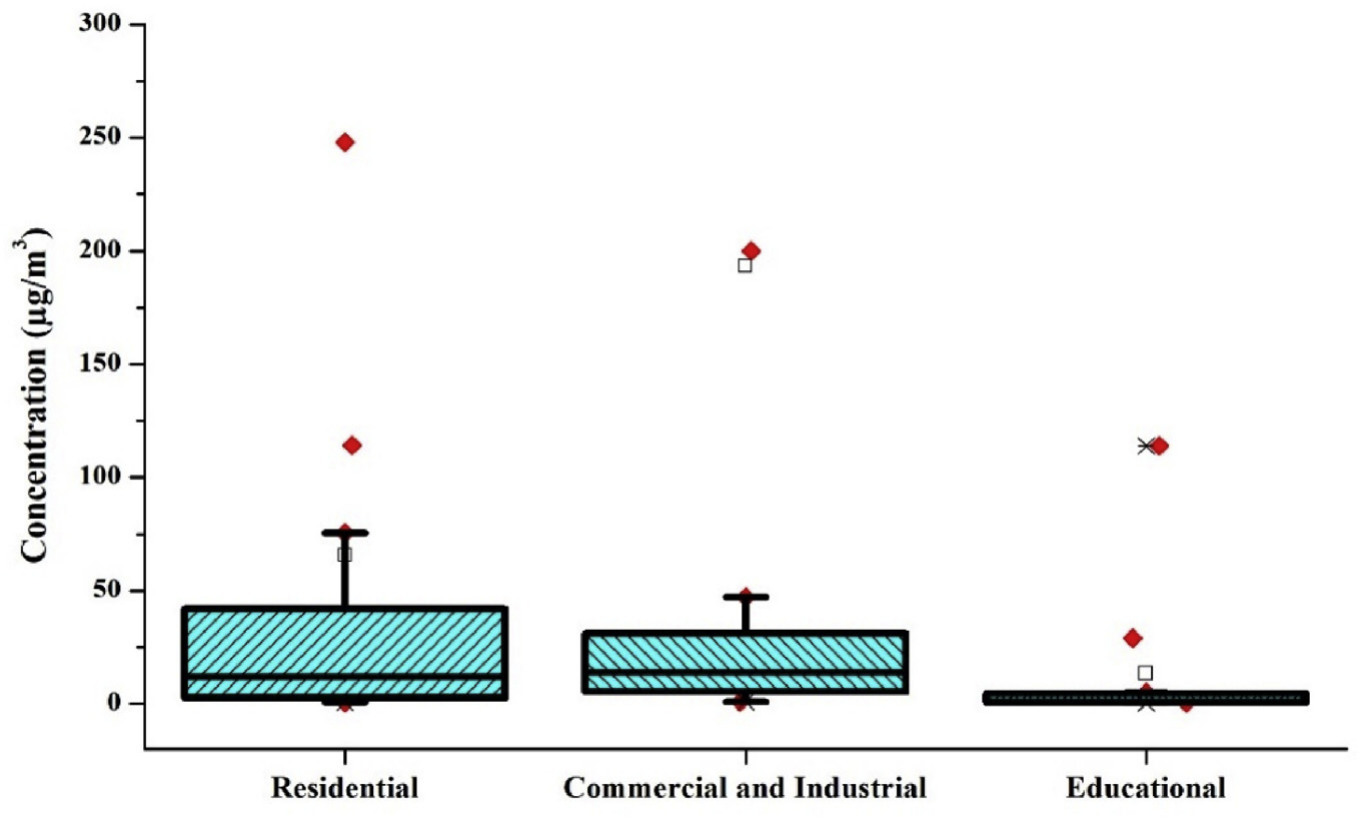
Box plot representing the distribution of benzene in Indoor environment.

**Figure 5:**
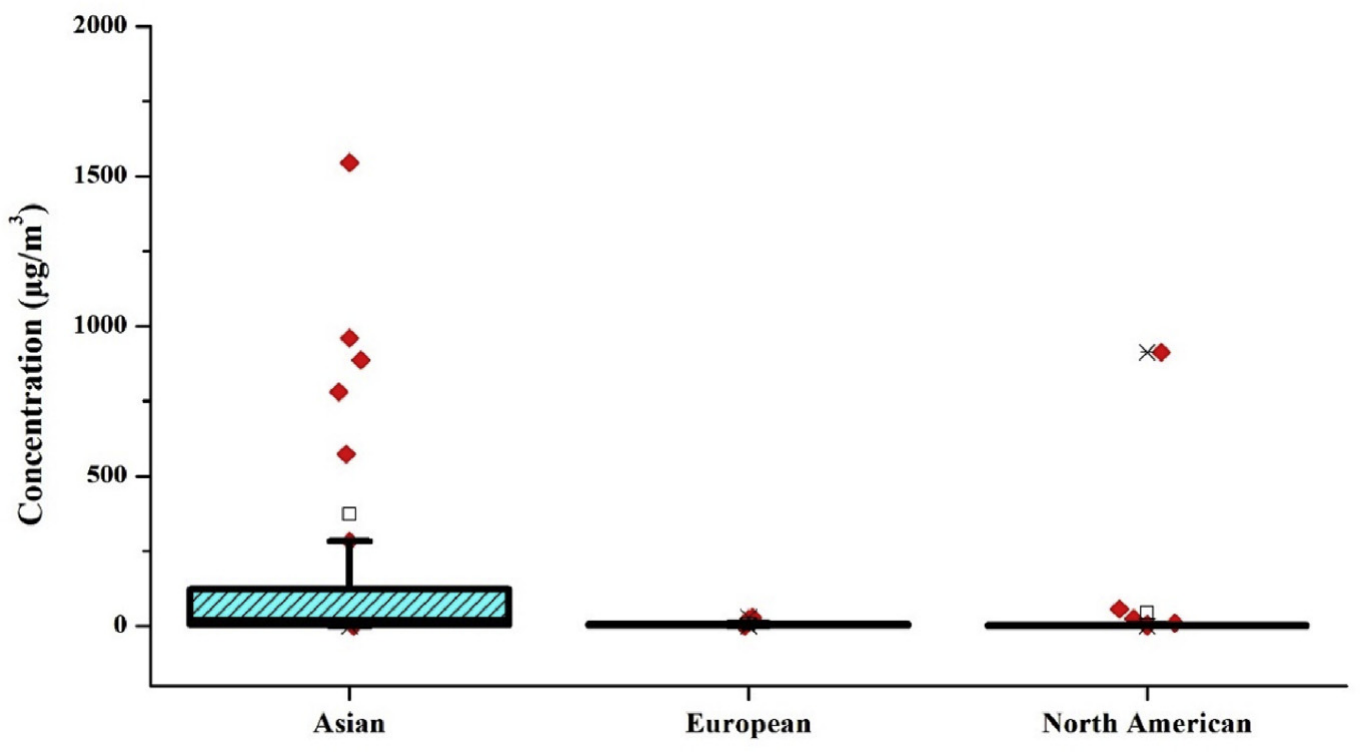
Box plot representing the distribution of benzene in an outdoor environment.

The average level of indoor benzene for different occupancy categories was calculated without considering the sampling duration. The distribution of benzene in these environments is represented by box plot using Origin Pro 8.5 (Figures 4 and 5). The average level of benzene was 78.4 ± 210 μg/m^3^, 22.45 ± 41.3 μg/m^3^ and 23.65 ± 31.4 μg/m^3^ in residential, commercial and Industrial & Educational establishments, respectively. In the case of ambient benzene levels, the average values of Asian, European and American countries were calculated. The mean benzene levels found were 371.4 ± 1566.7 μg/m^3^, 5.6 ± 6.6 μg/m^3^, 44.6 ± 189.6 μg/m^3^ in Asian, European and North American countries, respectively. The level of benzene found in the ambient air near to industrial regions across the globe was 479.65 ± 1050.14 μg/m^3^. Comparing the indoor and outdoor levels in Asian, European and North American countries the overall mean of outdoor concentrations was higher when compared with indoor benzene levels. It was found that in Asian countries the outdoor benzene level was approximately 3.5 times higher than the indoor benzene levels. Similarly, in the case of European countries, the outdoor concentrations were approximately 1.2 times higher than the indoor benzene concentrations. Highest Outdoor to Indoor ratio of 7.7 was obtained in the case of North American countries.

The concentrations of benzene collected in the literature search across Asian, European and North American countries were subjected to One-way ANOVA using SPSS software and the significance level was found to be 0.223, which is greater than 0.05. Thus, there is no significant difference between each group. A similar test was conducted for the level of benzene within residential, Commercial & Industrial and educational buildings and found that (p = 0.628) there is no significant difference in the amount of benzene in these microenvironments. Finally, the benzene concentration in outdoor and indoor air were statistically compared, and the results obtained was similar, i.e., there is no significant difference (p = 0.678) in the benzene levels.

## 7 Adequacy of benzene standards for health protection

According to ‘Human Health Evaluation Manual, Supplemental Guidance: Update of Standard Default Exposure Factors’ (US EPA, 2014), risk assessment was carried for all the permissible benzene standard values prescribed by different national legislation. As per the ‘Recommended Default Exposure Factors’ (2014), the average weight of the person was considered as 80 kg, outdoor exposure frequency as 225 days/year, lifetime as 70 years and air inhalation rate as 20 m^3^/day. By considering all the above factors Chronic Daily Intake (CDI) was determined (US EPA, 2014). Potency factor of 2.9 × 10^−2^ (mg/kg/day)^−1^ (Nazaroff & Lisa, 2000) was used to determine the lifetime incremental cancer risk. The reference concentration for inhalation exposure was considered to be 0.0085 mg/kg/day. Exposure length was taken as 0.17 (4 h/day), and exposure duration 25 years (Edokpolo et al., 2015) to calculate Life time Average Daily Dose (LADD) and Hazard Quotient (HQ). Table 4 shows the tabulation of the calculated risk associated with each permissible concentration.

**Table 4:** Risk calculated for available standard values of benzene.

Standard value in μg/m^3^	Incremental lifetime cancer risk (one in)	HQ	Country
20	31325	0.022016	Syria
10	62651	0.011008	Morocco, South Africa, Vietnam
5	125302	0.005504	European Union, India, Lebanon, Russia, South Korea, Botswana, Albania, Colombia
4	156628	0.004403	Peru
3.6	174031	0.003963	New Zealand
3	208837	0.003302	Iraq, Japan, Sweden, Malta
2	313256	0.0187133	France
1.3	481933	0.001431	Israel

As expected, the permissible values are not equally protective. HQ in all the cases was <1 suggesting acceptable risk for the exposed population in case of non-cancer risk. But, in the case of carcinogenic risk, considering one in 100 thousand as an acceptable incremental risk, values higher than 5 μg/m^3^ cannot be set as standard. Alternatively, if a higher level of protection, characterized by 1 in a million permissible risk is required, even the most stringent standard is insufficient.

## 8 Conclusion

Among the 193 countries, only 53 (≈27%) have a standard for benzene even when it is a chemical of concern and studies have shown its presence in the indoor and outdoor air throughout the world. The standards prescribed by legislations vary in more than one order of magnitude. Even adjoining countries have standards that are quite different. As air pollution does not respect political boundaries, such differences in standards will make the regulations meaningless. In addition to this, the available standards are not protective of human health. Since air pollution is a global issue there is an urgent need to harmonize the standards worldwide. Such a move will improve the air quality locally, nationally and globally. Along with that the governments should come forward to enact stricter clean air legislation and should carry out regular air quality monitoring.

Considering the studies carried out worldwide, the most commonly used method for the detection of benzene is sampling by sorbent tubes followed by analysis in GC-MS. This itself is the method most commonly used in Asian, European and American countries, taken separately. Thus, globally there is uniformity in the measurement techniques for benzene. But this technique gives only the average value for the entire monitoring period and not the instantaneous concentrations. This could prove a constraint in real-time monitoring studies intended to identify the source, fate and dynamics of benzene in the environment. Thus, there is a need for a cost-effective and efficient continuous real-time monitoring method for benzene.

From the concentrations obtained from the extensive literature study, it cannot be said that there is any significant difference in benzene concentrations across different continents. In Asian countries, most of the studies have been carried out in the urban ambient environment. Rural regions are no longer cleaner than urban areas; hence monitoring studies should be carried out in both rural and urban environments. Personal exposure studies in industrial and commercial environments are rare in Asian countries. Future studies can focus on these areas. Studies on the seasonal variation of benzene level can also be carried out.

## Declarations

### Author contribution statement

All authors listed have significantly contributed to the development and the writing of this article.

### Funding statement

This research did not receive any specific grant from funding agencies in the public, commercial, or not-for-profit sectors.

### Competing interest statement

The authors declare no conflict of interest.

### Additional information

No additional information is available for this paper.

## Appendix A Supplementary data

supplementry material 1 supplementry material 1
