# Supplementary material for: Analysis of benzene air quality standards, monitoring methods and concentrations in indoor and outdoor environment
Source: Heliyon. 2019 Nov 29;5(11):e02918. doi: 10.1016/j.heliyon.2019.e02918 (PMC6895577; doi:10.1016/j.heliyon.2019.e02918)
Supplement: supplementry material 1 [file mmc1.docx]

ANALYSIS OF BENZENE AIR QUALITY STANDARDS, MONITORING METHODS AND CONCENTRATIONS IN INDOOR AND OUTDOOR ENVIRONMENT

Abinaya Sekar^1^, George K.Varghese^1*^, M K Ravi Varma^2^

^1^Department of Civil Engineering, Environmental Engineering Lab, National Institute of Technology Calicut, India - 673601

^2^Department of Physics, Applied Optics and Instrumentation Lab, National Institute of Technology Calicut – 673601

*Corresponding Author: Email : [gkv@nitc.ac.in](mailto:gkv@nitc.ac.in); Tel.: +91- 495-2286206, +91-9447581218

SUPPLEMENTARY MATERIAL 1

List of Key Compounds in Global Air Quality Standards

| Si. No | NAME OF THE COUNRTY | STD FOR BENZENE | AIR QUALITY STANDARDS- KEY POLLUTANTS | REFERENCE |
| --- | --- | --- | --- | --- |
| 1 | Afghanistan | NO | WHO guidelines | (clean air asia, 2006) |
| 2 | Albania | YES | SO_2_, NO_2_, O_3_, PM10, PM2.5, Benzene,  Toluene, Xylene | (Environmental centre for Administration & Technology, 2008) |
| 3 | Algeria | NO | NO_2_, SO_2_, O_3_, PM10 | (Journal Officiel De La Republique Algerienne N° 01, 2006) |
| 4 | Andorra | EU | Limit value for the protection of human health | (Butlletí Oficial del Principat d ’ Andorra Reglament de control de la contaminació atmosfèrica Decret que modifica el reglament de control de la contaminació atmosfèrica Exposició de motius Butlletí Oficial del Principat d ’ Andorra, 2009) |
| 5 | Angola | N/A | N/A | N/A |
| 6 | Antigua and Barbuda | N/A | N/A | N/A |
| 7 | Argentina | NO | CO, NO_X_, SO_2_, O_3,_ PM10, Particulate sedimentable | (Boletin Oficial, 1973) |
| 8 | Armenia | YES- Russia | Maximum allowable concentration | (European Union, 2012) |
| 9 | Australia | NO | CO, Pb, NO_2_, PM10, PM2.5, O_3_, SO_2_ | (NEPM, 2016) |
| 10 | Austria | EU | Limit value for human health Protection | (EU Directive, 2008) |
| 11 | Azerbaijan | YES-Russia | Maximum allowable concentration | (UNEP, 2015)^1^ |
| 12 | Bahamas | NO | Pb, SO_2_, NO_2_, PM10, O_3_ | (The Environmental Planning And Protection Act , 2000) |
| 13 | Bahrain | NO | SO_2_, NO_2_, PM10 | Airlex Database ^4^ |
| 14 | Bangladesh | NO | Co, Pb, NO_x_, TSP, PM10, PM2.5, O3, SO_2_ | (Recommendations Regarding Revisions To The Bangladesh National Ambient Air Quality Standards, 2007) |
| 15 | Barbados |  | N/A | N/A |
| 16 | Belarus | YES | Pb, Benzene, SO_2_, PM2.5, PM10, O_3_, NO_2_, CO, Bap, Cd, As, Ni | (European Union, 2012) |
| 17 | Belgium | EU | Limit value for the protection of human health | (EU Directive, 2008) |
| 18 | Belize | N/A | N/A | N/A |
| 19 | Benin | NO | Pb, SO_2_, PM10, O_3_, NO_2_, CO, HC | (World bank document)^2^ |
| 20 | Bhutan | NO | Follows WHO Guidelines | (Clean Air Asia, 2006) |
| 21 | Bolivia | NO | Pb, SO_2_, PM10, O_3_, NO_2_, CO, Hg, Mn, Cd, As, Zn, TSP, Hcl, H_2_S | (UNEP, 2015)^3^ |
| 22 | Bosnia and Herzegovina | NO | SO_2_, NO_2_, NO_X_, PM10, Cd, O_3_, CO, Pb, Cd, Zn, ULC | (Službene novine FBiH’” broj:12/05 Na) |
| 23 | Botswana | YES | SO_2_, NO_2_, CO, PM10, O_3_, Pb, Benzene | (Modupe O. Akinola1, 2017) |
| 24 | Brazil | NO | TSP, Smoke, Inhalable particles, SO_2_, CO, O_3_, NO_2_ | (Controle Da Poluição Do Ar - Pronar, 1990) |
| 25 | Brunei | N/A | N/A | N/A |
| 26 | Bulgaria | EU | Limit value for the protection of human health | (EU Directive, 2008) |
| 27 | Burkina Faso |  | Pb, SO_2_, PM10, O_3_, NO_2_, CO | (World bank document)^2^ |
| 28 | Burundi | NO-EAC | SO_X_, NO_X,_ NO_2,_ SPM, RPM, PM2.5, Pb, CO, NMHC, TVOC, O_3_ | (East African Standard, 2010) |
| 29 | Cabo Verde | N/A | N/A | N/A |
| 30 | Cambodia (Check) | NO | CO, NO_2_, SO_2_, O_3_, TSP | (CAI-Asia, 2006) |
| 31 | Cameroon | N/A | N/A | N/A |
| 32 | Canada | NO | SO_2_, PM2.5, PM10, O_3_, NO_2_, CO | (Canadian Air quality Standards)^5^ |
| 33 | Central African Republic (CAR) | N/A | N/A | N/A |
| 34 | Chad | N/A | N/A | N/A |
| 35 | Chile | NO | SO_2_, O3, NO_2_, CO, PM10, PM2.5 | (UNEP, 2015)^6^ |
| 36 | China | NO | SO_2_, NO_2_, CO, O_3_, PM10, PM2.5, TSP, NO_x_, Pb, Bap | (GB 3095-2012)^7^ |
| 37 | Colombia | YES | NO2, SO2, O3, PM 10, PM 2.5, Benzene, Pb, Cd | (UNEP, 2015)^8^  Airlex database ^4^ |
| 38 | Congo | N/A | N/A | N/A |
| 39 | Comoros | N/A | N/A | N/A |
| 40 | Costa Rica | NO | Pb, SO_2_, H_2_S, HF, TSP, HCL, NH3 PM10, O_3_, NO_2_, CO | Airlex Database ^4^ |
| 41 | Cote d'Ivoire | N/A | N/A | N/A |
| 42 | Croatia | EU | Limit value for the protection of human health | (EU Directive, 2008) |
| 43 | Cuba | NO | Maximum allowable concentrations | Airlex Database ^4^ |
| 44 | Cyprus | EU | Limit value for the protection of human health | (EU Directive, 2008) |
| 45 | Czech Republic | EU | Limit value for the protection of human health | (EU Directive, 2008) |
| 46 | Democratic Republic of the Congo | N/A | N/A | N/A |
| 47 | Denmark | EU | Limit value for the protection of human health | (EU Directive, 2008) |
| 48 | Djibouti | N/A | N/A | N/A |
| 49 | Dominica | N/A | N/A | N/A |
| 50 | Dominican Republic | NO | TSP, Pb, SO_2_, PM2.5, PM10, O_3_, NO_2_, CO | Airlex Database ^4^ |
| 51 | Egypt | NO | Black smoke, TSP, PM10, PM2.5, SO_2_, CO, NO_2_, NO_X_, O3, Pb | (EPIQ, 2001) |
| 52 | Equatorial Guinea | N/A | N/A | N/A |
| 53 | Eritrea | N/A | N/A | N/A |
| 54 | Estonia | EU | Limit value for the protection of human health | (EU Directive, 2008,) |
| 55 | Ethiopia | N/A | N/A | N/A |
| 56 | Ecuador | NO | SO_2_, PM2.5, PM10, O_3_, NO_2_, CO | Airlex database ^4^ |
| 57 | El Salvador | NO | TSP, Pb, SO_2_, PM2.5, PM10, O_3_, NO_2_, CO | Airlex database ^4^ |
| 58 | Eswatini | N/A | N/A | N/A |
| 59 | Fiji | NO | CO, NO_2_, O_3_, SO_2_, PM10 | (FIJ : Transport Infrastructure Investment Sector Project, 2014) |
| 60 | Finland | EU | Limit value for the protection of human health | (EU Directive, 2008) |
| 61 | France | EU | Limit value for the protection of human health | (EU Directive, 2008) |
| 62 | Gabon | N/A | N/A | N/A |
| 63 | Gambia | NO | SO_2_, PM10, NO_2_, Pb | (NATIONAL ENVIRONMENT MANAGEMENT ACT, 1999) |
| 64 | Georgia | NO | Pb, SO_2_, O_3_, NO_2_, CO | Airlex Database ^4^ |
| 65 | Germany | EU | Limit value for the protection of human health | (EU Directive, 2008) |
| 66 | Ghana | NO | CO, NO2, SO2, PM 10 | Airlex Database ^4^ |
| 67 | Greece | EU | Limit value for the protection of human health | (EU Directive, 2008) |
| 68 | Grenada | N/A | N/A | N/A |
| 69 | Guatemala | NO | SO_2_, PM2.5, PM10, O_3_, NO_2_, CO | Airlex Database ^4^ |
| 70 | Guinea | N/A | N/A | N/A |
| 71 | Guinea-Bissau | N/A | N/A | N/A |
| 72 | Guyana | NO | Smoke, solid particles, Fluoride compounds, Hcl, Cl, H_2_S, NO_X_, CO | (Environmental Protection Act ,1996) |
| 73 | Haiti | N/A | N/A | N/A |
| 74 | Honduras | N/A | N/A | N/A |
| 75 | Hungary | EU | Limit value for the protection of human health | (EU Directive, 2008) |
| 76 | Iceland | YES-Benzene | Limit value for the protection of human health | (EU Directive, 2008) |
| 77 | India | YES | SO_2_, NO_2_, PM10, PM2.5, O3, Pb, CO, NH_3_, Benzene, BaP, As, Ni | (The Gazette of India,2009) |
| 78 | Indonesia | NO | SPM, PM10, SO_2_, NO_2_, O_3_, Pb, CO | (Indonesia : Air Quality Profile, 2010) |
| 79 | Iran | NO | Particulates, SO_X_, CO, Photochemical oxidants, HC, NO_X_ | (Engineering Standard For Air Pollution Control First Edition October, 2007) |
| 80 | Iraq | YES | CO, SO_2_, NO_2_, O_3_, PM10, PM2.5, TSP, Falling dust, HC, Pb | (Ambient air quality standards)^9^ |
| 81 | Ireland | EU | Limit value for the protection of human health | (EU Directive, 2008) |
| 82 | Israel | YES | Benzene, SO_2_, PM10, O_3_, NO_2_, CO | (Environmental Health in Israel, 2017) |
| 83 | Italy | EU | Limit value for the protection of human health | (EU Directive, 2008) |
| 84 | Jamaica | NO | TSP, Pb, SO_2_, PM10, O_3_, NO_2_, CO | (Ambient Air Quality Standards Regulations for Jamaica,1996) |
| 85 | Japan | YES | C.P:SO_2_, CO, SPM, NO_2_, Photochemical oxidants | (Japan Environmental Quality Standards, 2009)^10^ |
| 86 | Jordan | NO | Cd, NH3, TSP, Pb, PM 2.5, PM 10 | (Environmental & Social Assesment Overview, 2010) |
| 87 | Kazakhstan | NO | CO, NO_X_, NO_2_, Suspended dust, phenol, formaldehyde, Pb, NH_3_, SO_2_ | (No Title, 2015)^11^ |
| 88 | Kenya | NO | CO, NO_X_, NO_2_, Dust, Phenol, Formaldehyde, Pb, NH_3_, SO_2_, H_2_S, Cl, HF, Cu, HCl | (The Environmental Management And Co-Ordination Act, 1999) |
| 89 | Kiribati | N/A | N/A | N/A |
| 90 | Kuwait | NO | CO, NO_2_, SO_2_, O_3_, Pb, PM10, PM2.5 | (Article 76 – Ambient Air Quality, 2011)^12^ |
| 91 | Kyrgyzstan | NO | TSP, SO_2_, CO, NO_2_, NO, Tetra ethyl lead | (Environmental Assessment Report Draft, 2010) |
| 92 | Laos | N/A | N/A | N/A |
| 93 | Latvia | EU | Limit value for the protection of human health | (EU Directive, 2008) |
| 94 | Lebanon | YES | SO_2_, NO_2_, CO, O_3_, TSP, PM2.5, PM10, Pb, Benzene | (European Union, 2016) |
| 95 | Lesotho | N/A | N/A | N/A |
| 96 | Liberia | N/A | N/A | N/A |
| 97 | Libya | N/A | N/A | N/A |
| 98 | Liechtenstein | NO | Bap, Cd, As, Ni, SO_2_, PM10, O_3_, NO_2_, CO, Pb | Airlex database ^4^ |
| 99 | Lithuania | EU | Limit value for the protection of human health | (EU Directive, 2008) |
| 100 | Luxembourg | EU | Limit value for the protection of human health | (EU Directive, 2008) |
| 101 | Macedonia (FYROM) | YES-EU | Limit value for the protection of human health | (EU Directive, 2008) |
| 102 | Madagascar | N/A | N/A | N/A |
| 103 | Malawi | NO | PM10, PM2.5, CO, SO_2_, NO_2_, O_3_, Pb, Photochemical oxidants | (Wilson, Mapoma, Tenthani, & Tsakama, 2014) |
| 104 | Malaysia | NO | PM10, PM2.5, SO_2_, NO_2_, O_3_, CO | (New Malaysia Ambient Air Quality Standard)^13^ |
| 105 | Maldives | N/A | N/A | N/A |
| 106 | Mali | N/A | N/A | N/A |
| 107 | Malta | EU | Limit value for the protection of human health | (EU Directive, 2008) |
| 108 | Marshall Islands | N/A | N/A | N/A |
| 109 | Mauritania | N/A | N/A | N/A |
| 110 | Mauritius | Mauritius | TSP, PM10, SO_2_, NO_2_, CO, Pb, O_3_ | (Ambient Air Quality and Emission Standards for Mauritius, 2011) |
| 111 | Mexico | NO | Pb, SO_2_, PM10, O_3_, NO_2_, CO | Airlex Database ^4^ |
| 112 | Micronesia | N/A | N/A | N/A |
| 113 | Moldova | YES-RUSSIA | Maximum allowable Concentrations | (European Union, 2012) |
| 114 | Monaco | N/A | N/A | N/A |
| 115 | Mongolia | NO | SO_2_, NO_2_, TSP, O_3_, Pb, BaP | (МОНГОЛ УЛСЫН СТАНДАРТ Ангилалтын, 2008) |
| 116 | Montenegro | YES-EU | Limit value for the protection of human health | (EU Directive, 2008) |
| 117 | Morocco | YES | SO_2_, NO_2_, CO, PM10, O_3_, benzene | (Chirmata, Leghrib, & Ichou, 2017) |
| 118 | Mozambique | NO | SO_2_, NO_2_, CO, O_3_, TSP, Pb | (Baobab Tete Iron Ore Project , Mozambique, 2015) |
| 119 | Myanmar (Burma) | N/A | N/A | N/A |
| 120 | Namibia | N/A | N/A | N/A |
| 121 | Nauru | N/A | N/A | N/A |
| 122 | Nepal | NO | TSP, PM10, PM2.5, NO_2_, SO_2_, O_3_, CO, Pb | (Air Quality Management Action Plan for Kathmandu Valley Quest Forum Pvt . Ltd , 2017) |
| 123 | Netherlands | EU | Limit value for the protection of human health | (EU Directive, 2008) |
| 124 | New Zealand | YES | Benzene, SO_2_, PM10, O_3_, NO_2_, CO | (Ministry for the Environment and the Ministry of Health, 2002) |
| 125 | Nicaragua | NO | Pb, TSP, SO_2_, PM10, O_3_, NO_2_, CO | Airlex Database ^4^ |
| 126 | Niger | N/A | N/A | N/A |
| 127 | Nigeria | NO | Particulates, SO_2_, NMHC, CO, NO_X_, Photochemical oxidant | (ADOKI, 2012) |
| 128 | North Korea | YES-EU | Limit value for the protection of human health | (EU Directive, 2008) |
| 129 | Norway | YES-EU | Limit value for the protection of human health | (EU Directive, 2008) |
| 130 | Oman | NO | NO_2_, SO_2_, CO, H_2_S, O_3_, HCNM, PM10 | Airlex Database ^4^ |
| 131 | Pakistan | NO | SO_2_, NO, NO_2_, O_3_, SPM, PM10, PM2.5, Pb, CO | (Clean Air Asia, 2006) |
| 132 | Palau | NO | SO_2_, PM, CO, Photochemical oxidants, HC, NO_X_ | (Environmental quality Protection Board, 1999) |
| 133 | Panama | NO | SO_2_, PM10, O_3_, NO_2_, CO | Airlex database ^4^ |
| 134 | Papua New Guinea | NO | SO_2_, NO_2_, CO, H_2_S, PM10, PM2.5, TSP, O_3_ | (Papua New Guinea LNG Project Environmental Standards, 2010) |
| 135 | Paraguay | N/A | N/A | N/A |
| 136 | Peru | YES | Pb, Benzene, SO_2_, PM2.5, PM10, O_3_, NO_2_, CO | (Decreto Supremo N 074 2001-Pcm, 2007) |
| 137 | Philippines | NO | TSP, PM10, PM2.5, SO_2_, NO_2_, O_3_, CO, Pb | (Air Quality In The Philippines , 2015) |
| 138 | Poland | EU | Limit value for the protection of human health | (EU Directive, 2008) |
| 139 | Portugal | EU | Limit value for the protection of human health | (EU Directive, 2008) |
| 140 | Qatar | NO | SO_2_, NO_2_, O_3_, CO, PM10, PM2.5 | (State of Qatar, 2014) |
| 141 | Romania | EU | Limit value for the protection of human health | (EU Directive, 2008) |
| 142 | Russia | YES | CO, H_2_S, NO_2_, NO, SO_2_, Alkanes, Pentanes, hexane, Benzene, Toluene, Xylene, O_3_, PM10, PM2.5 | (Project Environmental And Social Standards, 2014) |
| 143 | Rwanda | NO | Pb, TSP, SO_2_, PM2.5, PM10, O_3_, NO_2_, CO | Airlex database ^4^ |
| 144 | Saint Kitts and Nevis | N/A | N/A | N/A |
| 145 | Saint Lucia | N/A | N/A | N/A |
| 146 | Saint Vincent and the Grenadines | N/A | N/A | N/A |
| 147 | Samoa | NO | SO_2_, PM10, O_3_, NO_2_, Pb, CO | Airlex database ^4^ |
| 148 | San Marino | N/A | N/A | N/A |
| 149 | Sao Tome and Principe | N/A | N/A | N/A |
| 150 | Saudi Arabia | NO | SO_2_, O_3_, NO_2_, CO, H_2_S, F | (Kingdom of Saudi Arabia Presidency of Meteorology and Environment, 2001) |
| 151 | Senegal | NO | Cd, Pb, CO, NO_2_, SO_2_, PM10, O_3_ | (Norme Senegalaise, 2003) |
| 152 | Serbia | YES-EU | Limit value for the protection of human health | (EU Directive, 2008) |
| 153 | Seychelles | N/A | N/A | N/A |
| 154 | Sierra Leone | N/A | N/A | N/A |
| 155 | Singapore | NO | SO_2_, PM2.5, PM10, O_3_, NO_2_, CO | (Clean Air Asia, 2006) |
| 156 | Slovakia | EU | Limit value for the protection of human health | (EU Directive, 2008) |
| 157 | Slovenia | EU | Limit value for the protection of human health | (EU Directive, 2008) |
| 158 | Solomon Islands | N/A | N/A | N/A |
| 159 | Somalia | N/A | N/A | N/A |
| 160 | South Africa | YES | SO_2_, NO_2_, PM10, O_3_, Benzene, Pb, CO | (Department of Environmental Affairs South Africa, 2009) |
| 161 | South Korea | YES | SO_2_, CO, NO_2_, PM10, O_3_, Pb, Benzene, | (Ambient Air Quality Standards)^14^ |
| 162 | South Sudan | N/A | N/A | N/A |
| 163 | Spain | EU | Limit value for the protection of human health | (EU Directive, 2008) |
| 164 | Sri Lanka | NO | CO, NO_2_, SO_2_, O_3_, Pb, SPM | (Progress on Environmental Quality, 2008) |
| 165 | Sudan | N/A | N/A | N/A |
| 166 | Suriname | N/A | N/A | N/A |
| 167 | Sweden | EU | Limit value for the protection of human health | (EU Directive, 2008) |
| 168 | Switzerland | NO | Pb, Cd, CO, PM 10, SO_2_, NO_2_, O_3_ | (Luftreinhalte-Verordnung, 2010) |
| 169 | Syria | YES | SO_2_, NO_2_, O_3_, CO, Pb, TSP, PM10, Benzene | (National Air quality standards syria ) |
| 170 | Tajikistan | YES-Russia | Maximum allowable concentration | (Tajikistan : CAREC Corridor 3 ( Dushanbe – Uzbekistan Border ) Improvement Project, 2010) |
| 171 | Tanzania | NO | PM 10, SO_2_, Pb, CO, O_3_, NO_2_ | Airlex database ^4^ |
| 172 | Thailand | NO | CO, NO_2_, SO_2_, O_3_, TSP, PM10, Pb, PM2.5,9 VOCs | (National Ambient Air Quality Standards ( NAAQSs ) in Thailand) |
| 173 | Timor-Leste | N/A | N/A | N/A |
| 174 | Togo | N/A | N/A | N/A |
| 175 | Tonga | N/A | N/A | N/A |
| 176 | Trinidad and Tobago | NO | Pb, HF, Hg, CO, PM 2.5, H_2_S, NO_2_, PM 10, SO_2_, Hcl, O_3_, TSP, Cd | Airlex Database ^4^ |
| 177 | Tunisia | N/A | N/A | N/A |
| 178 | Turkey | YES-EU | Limit value for the protection of human health | (EU Directive, 2008) |
| 179 | Turkmenistan | NO | TSP, PM10, PM2.5, SO_2_, NO_2_, NO, CO, O_3_, Formaldehyde, Phenol, Cl, NH_3_, H_2_S, HF | (Environmental Performance Reviews,2012) |
| 180 | Tuvalu | N/A | N/A | N/A |
| 181 | Uganda | NO-EAC | SO_X_, NO_X,_ NO_2,_ SPM, RPM, PM2.5, Pb, CO, NMHC, TVOC, O_3_ | (East African Standard, 2010) |
| 182 | Ukraine | YES-Russia | Maximum allowable Concentration | (European Union, 2012) |
| 183 | United Arab Emirates (UAE) | NO | SO_2_, CO, NO_2_, O_3_, TSP, PM10 | (Regulation EN – 4 . 0 Goverment of Dubai,2013) |
| 184 | United Kingdom (UK) | EU | Limit value for the protection of human health | (EU Directive, 2008) |
| 185 | United States of America (USA) | NO | O_3_, Pb, SO_2_, CO, PM 2.5, PM 10, NO_2_ | (National Ambient Air Quality Standards ( NAAQS ) US EPA, 2015) |
| 186 | Uruguay | N/A | N/A | N/A |
| 187 | Uzbekistan | NO | Dust, NOx, SO_2_, O_3_ | Airlex database ^4^ |
| 188 | Vanuatu | N/A | N/A | N/A |
| 189 | Venezuela | NO | CO, PM10, SO_2_, NO_2_, O_3_ | (Ministerio del Ambiente y Recursos Naturales Instituto Nacional de Parques (INPARQUES),1995) |
| 190 | Vietnam | YES | SO_2_, CO, NO_2_, O_3_, TSP, PM10, Pb, Benzene, toluene, xylene | (TCVN 5938, 2005) |
| 191 | Zambia | NO | TSP, PM10, NO_X_, CO, Pb | (The Environmental Protection And Pollution Control Act, 1994) |
| 192 | Zimbabwe | NO | CO, Pb, NO_2_, O_3_, PM10, PM2.5, SO_2_ | (Standards Association Of Zimbabwe,2014) |
| 193 | yemen | N/A | N/A | N/A |

Reference

1. European Parliament, Directive 2008/50/EC Of the European Parliament and Of the Council Of 21 May 2008 on Ambient Air Quality and Clear Air for Europe, 2008, Official Journal of The European Union.

<https://eur-lex.europa.eu/LexUriServ/LexUriServ.do?uri=OJ:L:2008:152:0001:0044:en:PDF>

1. Clean Air Asia (CAI-Asia), Country Synthesis report on urban Air quality Management Afghanistan, Discussion Draft December 2006.

<http://cleanairasia.org/wp-content/uploads/portal/files/documents/afghanistan_0.pdf>

1. Tirana Air Quality Report, Environmental Centre for administration & Technology (ECAT), December 2008.

<http://ec.europa.eu/environment/life/project/Projects/index.cfm?fuseaction=home.showFile&rep=file&fil=SUSTRAFFTIA_Tirana_air_Quality_Report.pdf>

1. Journal Officiel De La Republique Algerienne N° 01, 8 Dhou El Hidja 1426, 8 Janvier 2006.

<http://www.sante.dz/jms2010/oms/dec06-03.pdf>

1. Butlletí Oficial del Principat d’Andorra, Reglament de control de la contaminació atmosfèrica, Núm. 17 any 21 -4.3.2009.

<http://airlex.web.ua.pt/uploads/standards/06200905_bop21017.pdf>

1. Ley Nacional 20.284, BOLETIN OFICIAL - 03/05/1973

<http://www.prosap.gov.ar/docs/UAS-20284ContaminacionAtmosferica.pdf>

1. Air Quality Governance in ENPI East Countries, General System Gap Analysis, August 2012.

<https://europa.eu/capacity4dev/file/21481/download?token=nbBtC_N_>

1. National Environment Protection (Ambient Air Quality) Measure, The Office of Legislative Drafting, Attorney-General’s Department, Canberra, July 2003.

<https://www.legislation.gov.au/Details/C2004H03935>

1. <https://wedocs.unep.org/bitstream/handle/20.500.11822/17060/Azerbaijan.pdf?sequence=1&isAllowed=y> ^1^
2. The Environmental Planning and Protection Act Of 2000, Pollution Control and Waste Management Regulations Bahamas

<http://www.best.gov.bs/Documents/Draft-PollutionControl+WasteMgmt%20regulations.pdf>

1. Recommendations Regarding Revisions to the Bangladesh National Ambient Air Quality Standards

<http://case.doe.gov.bd/file_zone/feedback/Revision%20of%20National%20Ambient%20Air%20Quality%20Standard.pdf>

1. Air Quality Governance in ENPI East Countries Inception Report

<https://europa.eu/capacity4dev/file/21481/download?token=nbBtC_N_>

1. [http://documents.worldbank.org/curated/en/936031468000276054/text/677940WP0P07690020120Box367897B0ACS.txt  ^2^](http://documents.worldbank.org/curated/en/936031468000276054/text/677940WP0P07690020120Box367897B0ACS.txt%20%202)
2. Clean Air Asia (CAI-Asia), Country Synthesis report on urban Air quality Management Bhutan, Discussion Draft December 2006.

<http://cleanairasia.org/wp-content/uploads/portal/files/documents/bhutan_0.pdf>

1. <https://wedocs.unep.org/bitstream/handle/20.500.11822/17151/Bolivia.pdf?sequence=1&isAllowed=y> ^3^
2. ''Službene novine FBiH'' broj:12/05, Na osnovu člana 27.stav 1. i člana 38. stav 2. Zakona o zaštiti zraka ("Službene novine Federacije BiH", broj 33/03), federalni ministar prostornog uređenja i okoliša donosi

<http://extwprlegs1.fao.org/docs/pdf/bih149098.pdf>

1. Modupe O. Akinola1, M. L. and E. O. D. (2017). THE CLEAN AIR JOURNAL Air quality management in Botswana, *27*(1), 1–<http://www.cleanairjournal.org.za/download/volume27_no1_2017_oe03.pdf>
2. RESOLUÇÃO CONAMA nº 3, de 28 de junho de 1990, Publicada no DOU, de 22 de agosto de 1990, Seção 1, páginas 15937-15939

<http://www.ibram.df.gov.br/images/resol_03.pdf>

1. East African Air Quality standard, CD/T/66/2010, ICS 87. 040

eac-quality.net/fileadmin/rwanda/user.../EAS-Air_Quality-Specification_01.doc

1. Clean Air Asia (CAI-Asia), Country Synthesis report on urban Air quality Management Combodia, Discussion Draft December 2006.

<http://cleanairasia.org/wp-content/uploads/portal/files/documents/cambodia_0.pdf>

1. <http://airlex.web.ua.pt/airlexDatabasePublic> ^4^
2. <http://www.ec.gc.ca/default.asp?lang=En&n=56D4043B-1&news=A4B2C28A-2DFB-4BF4-8777-ADF29B4360BD> ^5^
3. <https://wedocs.unep.org/bitstream/handle/20.500.11822/17166/Chile.pdf?sequence=1&isAllowed=y> ^6^
4. <https://www.transportpolicy.net/standard/china-air-quality-standards/> ^7^
5. <https://wedocs.unep.org/bitstream/handle/20.500.11822/17168/Colombia.pdf?sequence=1&isAllowed=y> ^8^
6. Alan P. Leob, Esq, Dr. Mahmoud M.Nasralla, Review and Assessment of Air Quality standards in Egypt, May 2001.

<https://rmportal.net/.../environmental-policy.../egyptian-environmental-policy-progra>...

1. Environmental Assessment and Review Framework, Transport Infrastructure Investment Sector Project (RRP FIJ 48141), 2014

<https://www.adb.org/sites/default/files/linked-documents/48141-001-earfab.pdf>

1. Environmental Quality Standards Regulations, 1999.

<http://extwprlegs1.fao.org/docs/pdf/gam95812.pdf>

1. Environmental Protection Act, 1996

<http://extwprlegs1.fao.org/docs/pdf/guy121168.pdf>

1. National Ambient Air Quality Standards, The gazette of India, 2009

<http://www.moef.nic.in/sites/default/files/notification/Recved%20national.pdf>

1. Indonesia Air quality Profile, Clean Air Asia, 2010 Edition

<http://www.indiaenvironmentportal.org.in/files/Indonesia_Air_Quality_Profile_-_2010_Edition.pdf>

1. Engineering standard for Air pollution Control, IPS-E-SF-860(1), 2007

<http://igs.nigc.ir/STANDS/IPS/e-sf-860.PDF>

1. <http://www.moo.oil.gov.iq/Envirnment/PDF%20files/14.pdf> ^9^
2. Environmental heath in Israel, 2017

<https://www.health.gov.il/PublicationsFiles/BSV_sviva2017_EN.pdf>

1. Ambient Air Quality Standards Regulations for Jamaica - August 1996

<http://nepa.gov.jm/standards/air_quality_standards_regulations.pdf>

1. [https://www.env.go.jp/en/air/aq/aq.html ^10^](https://www.env.go.jp/en/air/aq/aq.html%2010)
2. Feasibility study, environmental and social impact assessment and detailed designs and bidding documents for zarqa governorate waste water system reinforcement and expansion project environmental and social assessment overview, 2010.

<http://www.mca-jordan.gov.jo/SystemFiles/Pages/file_635041052815291862.pdf>

1. <https://www.env.go.jp/earth/coop/coop/c_report/kazakhstan_h17/english/pdf/008.pdf> ^11^
2. Environmental Management and Co-ordination (Conservation of Biological Diversity and Resources, Access to Genetic Resources and Benefit Sharing) Regulations, 2006.

<http://www.wipo.int/edocs/lexdocs/laws/en/ke/ke013en.pdf>

1. <http://www.assekuwait.org/download/books/3112141316477667.pdf> ^12^
2. Environmental Assessment Report, KGZ: CAREC Transport Corridor 1 (Bishkek–Torugart Road) Project 3

<https://www.adb.org/sites/default/files/linked-documents/42399-02-kgz-eiaab.pdf>

1. Support to Reforms – Environmental Governance, Beirut, Lebanon, European Union – ENPI/2014/337-755, 2014

<http://www.moe.gov.lb/getattachment/88e45b95-bfc4-4f6c-b261-c10a26d3ba22/.aspx>

1. Wilson, H., Mapoma, T., Tenthani, C., & Tsakama, M. (2014). Air quality assessment of carbon monoxide , nitrogen dioxide and sulfur dioxide levels in Blantyre , Malawi : a statistical approach to a stationary environmental monitoring station, *8*, 330–343.

https://doi.org/10.5897/AJEST2014.1696

1. <https://www.doe.gov.my/portalv1/wp-content/uploads/2013/01/Air-Quality-Standard-BI.pdf> ^13^
2. Ambient Air Quality and Emission Standards for Mauritius, Appendix C, Conversion of tank for oil storage, 2011

<http://environment.govmu.org/English/eia/Documents/Reports/petro_storage/SOFT%20COPY%20MMCO%20EIA/air%20qua.pdf>

1. Air Quality Governance in ENPI East Countries, EuropeAid/129522/C/SER/Multi Contract number 2010/232-231, GENERAL SYSTEM GAP ANALYSIS, 2012.

<https://europa.eu/capacity4dev/file/21481/download?token=nbBtC_N_>

1. МОНГОЛ УЛСЫН СТАНДАРТ, Ангилалтын код 13.040.01, 2007 <http://agaar.mn/files/article/23/%D0%90%D0%B3%D0%B0%D0%B0%D1%80%D1%8B%D0%BD%20%D1%87%D0%B0%D0%BD%D0%B0%D1%80%D1%8B%D0%BD%20%D1%81%D1%82%D0%B0%D0%BD%D0%B4%D0%B0%D1%80%D1%82-4585-2007..pdf>
2. Chirmata, A., Leghrib, R., & Ichou, I. A. (2017). Implementation of the Air Quality Monitoring Network at Agadir City in Morocco, 540–567. https://doi.org/10.4236/jep.2017.84037
3. Baobab Tete Iron Ore Project , Mozambique, Air quality Impact Assesment, 2015.

<http://www.cesnet.co.za/pubdocs/Baobab%20Tete%20Iron%20Ore%20ENGLISH%20CB127_180115/Air%20Quality%20Assessment.pdf>

1. Air Quality Management Action Plan for Kathmandu Valley, Ministry of Population and Environment, Department of Environment (Doenv) Sahidsukra Marg, Kupondole, Lalitpur, 2017

http://doenv.gov.np/files/download/Report%20on%20AQM%20Action%20Plan%202017.pdf

1. Ambient Air Quality Guidelines, Ministry for the Environment, New Zealand 2002

<https://www.mfe.govt.nz/sites/default/files/ambient-guide-may02.pdf>

1. ADOKI, A. (2012). Air Quality Survey of some locations in the Niger Delta Area. *J. Appl. Sci. Environ. Manage*, *16*(1), 125–134.

<http://www.bioline.org.br/pdf?ja12022>

1. Clean Air Asia (CAI-Asia), Country Synthesis report on urban Air quality Management Pakistan, Discussion Draft December 2006.

<http://cleanairasia.org/wp-content/uploads/portal/files/documents/pakistan_0.pdf>

1. The Republic of Palau Air Pollution Control Regulations, Chapter 2401-71

<http://extwprlegs1.fao.org/docs/pdf/pau42612.pdf>

1. Papua New Guinea LNG Project, Environmental Standards, PGGP-EH-SSZZZ-000002, 2010

[https://pnglng.com/media/PNG-LNG-Media/Files/Environment/Construction%20ESMP/PGGP-EH-SPENV- 000018-033_Env_Standards_Rev_1.pdf](https://pnglng.com/media/PNG-LNG-Media/Files/Environment/Construction%20ESMP/PGGP-EH-SPENV-%20%20000018-033_Env_Standards_Rev_1.pdf)

1. Decreto Supremo N° 074-2001-Pcm Reglamento De Estandares Nacionales De Calidad Ambiental Del Aire

<http://www.digesa.minsa.gob.pe/norma_consulta/DS-074-2001-PCM.pdf>

1. National Air quality status, 2008- 2015.

<http://emb.gov.ph/wp-content/uploads/2015/09/1-Air-Quality-1.8-National-Air-Quality-Status-Report-2008-2015.pdf>

1. State of Qatar, Ministry of Development Planning and Statistics, Environment Statistics Annual Report, 2013

<https://www.mdps.gov.qa/en/knowledge/Publications/Environment/Env_Environmental_Statistic_Report_En_2013.pdf>

1. Project Environmental and Social Standards, Appendix 2, 2014.

<http://yamallng.ru/Annex%202.%20ENG%20PDF%20Environmental%20and%20Social%20Standards%20Final%20Issue%2011%20Clean.pdf>

1. General Environmental Regulations and Rules for Implementation, Kingdom of Saudi Arabia Presidency of Meteorology and Environment, 2001

<https://www.pme.gov.sa/Ar/DataLists//DocumentLibrary/%D8%A7%D9%84%D9%86%D8%B8%D8%A7%D9%85%20%D8%A7%D9%84%D8%B9%D8%A7%D9%85%20%D9%84%D9%84%D8%A8%D9%8A%D8%A6%D8%A9%20%D9%88%D8%A7%D9%84%D9%84%D9%88%D8%A7%D8%A6%D8%AD%20%D8%A7%D9%84%D8%AA%D9%86%D9%81%D9%8A%D8%B0%D9%8A%D8%A9/General%20Environmental%20Regulations.pdf>

1. Norme Senegalaise Ns 05-062, Octobre 2003.

<http://extwprlegs1.fao.org/docs/pdf/sen54266.pdf>

1. Clean Air Asia (CAI-Asia), Country Synthesis report on urban Air quality Management Singapore, Discussion Draft December 2006

<http://cleanairasia.org/wp-content/uploads/portal/files/documents/singapore_0.pdf>

1. National Environmental Management: Air Quality Act, South Africa, 2004.

<https://www.environment.gov.za/sites/default/files/legislations/nemaqa_airquality_g32816gon1210.pdf>

1. <https://www.transportpolicy.net/standard/south-korea-air-quality-standards/> ^14^
2. Clean Air in Sri Lanka: Summary of progress on improving air quality, 2008.

<http://www.indiaenvironmentportal.org.in/files/SriLanka.pdf>

1. Luftreinhalte-Verordnung (LRV), 814.318.142.1, vom 16. Dezember 1985 (Stand am 1. Juni 2018).

<https://www.admin.ch/opc/de/classified-compilation/19850321/201806010000/814.318.142.1.pdf>

1. Environmental Impact Assessment Project Number: 42052 Tajikistan: CAREC Corridor 3 (Dushanbe–Uzbekistan Border) Improvement Project, 2010

<https://www.adb.org/sites/default/files/project-document/62861/42052-02-taj-eia.pdf>

1. Ambient Air Quality Standards Syria

<http://extwprlegs1.fao.org/docs/pdf/syr53691.pdf>

1. National Ambient Air Quality Standards (NAAQSs) in Thailand, Air Quality and Noise Management Bureau Pollution Control Department.

<http://infofile.pcd.go.th/air/cri170353_sec3.pdf>

1. United Nations Economic Commission for Europe, Turkmenistan, Environmental Performance Reviews, 2012 <http://www.zaragoza.es/contenidos/medioambiente/onu/941-eng.pdf>
2. Air Quality Governance in ENPI East Countries, EuropeAid/129522/C/SER/Multi Contract number 2010/232-231

<https://europa.eu/capacity4dev/file/21481/download?token=nbBtC_N_>

1. (Regulation EN – 4 . 0 Goverment of Dubai,2013

<http://trakhees.ae/en/ehs/env/Documents/Regulations/Regulation-4.0.pdf>

1. National Ambient Air Quality Standards ( NAAQS ), US EPA, 2015

https://www.epa.gov/sites/production/files/2015-02/documents/criteria.pdf

1. Maximum allowable concentration of hazardous substance in ambient air, TCVN5938-2005

<http://www.gree-vn.com/pdf/TCVN5938-2005.pdf>

1. Ministerio del Ambiente y Recursos Naturales Instituto Nacional de Parques (INPARQUES),1995

<http://www.cipram.com.ve/pdf/Decreto%20638%20Normas%20sobre%20calidad%20del%20Aire%20y%20Control%20de%20la%20Contaminacion%20Atmosferica.pdf>

1. The Environmental Protection and Pollution Control Act, Republic of Zambia, 1994

<http://www.vertic.org/media/National%20Legislation/Zambia/ZM_Environmental_Protection_Act.pdf>

1. Environmental Management Act, Government of Zimbabwe, Chapter 20:27, 2006

http://www.ilo.org/dyn/natlex/docs/ELECTRONIC/72825/95636/F-751709490/ZWE72825.pdf
